# Supplementary material for: Heavy-element production in a compact object merger observed by JWST
Source: Nature. 2023 Oct 25;626(8000):737–41. doi: 10.1038/s41586-023-06759-1 (PMC10881391; doi:10.1038/s41586-023-06759-1)
Supplement: Supplementary file 1 — The Supplementary Information contains further discussion relevant to the nature and properties of GRB 230307A. In particular, we place the burst properties in context with those of other GRBs in both the prompt (SI 1.1) and afterglow regimes (SI 1.2). We also provide further details on identifying the host galaxy and its detailed properties in SI 1.3 and 1.4. SI 1.5 considers the extreme brightness of the burst and investigates similarities and differences with other very bright GRBs. In SI 2, we consider the inferred event rates for bursts such as GRB 230307A. SI 3 provides further details on modelling the light curve (SI 3.1) and spectrum (SI 3.2). Finally, SI 4 investigates a broad range of alternative progenitor possibilities and their challenges and a justification for our chosen interpretation. This includes the high-z case (SI 4.1), lower-redshift supernovae (SI 4.2 and 4.3), Galactic sources (SI 4.4) and white dwarf–neutron star mergers (SI 4.5). Supplementary figures and tables are also provided as referenced in the main text, Methods and Supplementary Information. [file 41586_2023_6759_MOESM1_ESM.pdf]

nature portfolio

https://doi.org/10.1038/s41586-023-06759-1

Supplementary information

Heavy-element production in a compact object merger observed by JWST

In the format provided by the authors and unedited

Supplementary Information

This document contains supplementary information for the paper "Heavy element production in a compact object merger observed by JWST". While our data analysis steps are described in the Methods section. Here we provide additional discussion regarding the host galaxy properties, spectral modelling and the various progenitors considered for the emission in GRB 230307A.

SI.1 GRB 230307A in context

SI.1.1 Prompt emission

GRB 230307A is an exceptionally bright GRB. It has the second highest fluence of any GRB observed in more than 50 years of GRB observations[1]. While it remains a factor of 50 less fluent than GRB 221009A, it is still a factor ~ 2 brighter than GRB 130427A, the third brightest burst. Bursts with these extreme fluences are rare. In Figure 2, we plot the distribution of observed fluence for *Fermi*/GBM detected bursts. At the brighter end, the slope of the distribution is consistent with the expected $-3/2$ slope for a uniform distribution of sources. The extrapolation of this relation suggests that bursts like GRB 230307A should occur once every several decades. Notably, three bursts well above the extrapolation (GRB 130427A, GRB 230307A, GRB 221009A) may indicate that bright bursts arise more frequently than expected. However, observationally it is clear that GRB 230307A is, at least, a once-per-decade event.

The prompt light curve of GRB 230307A (Figure 1, main article) shows two distinct emission features: an initial episode of hard emission from the trigger until ≈ 18 s, then a softer episode from ≈ 19 s onwards. These distinct episodes of hard and soft emission are strongly reminiscent of the long-duration merger GRB 211211A, but the initial pulse complex is ~ 50 per cent longer in GRB 230307A when compared to the ~ 12 s duration seen in GRB 211211A[2]. The relative durations of the initial pulse complex in the two GRBs bear a striking resemblance to their relative time-averaged peak energies (936 ± 3 keV vs 647 ± 8 keV;[3,4]). In GRB 211211A, substantial spectral evolution was seen to drive the light curve, and the underlying radiation mechanism was identified as fast-cooling synchrotron emission[5]. The coherent development of the hardness ratio (lower panel of Figure 1, main article) indicated similar spectral evolution in GRB 230307A, which the spectral analysis confirmed. Indeed, as described in the Methods, the time-resolved spectral analysis of the prompt emission revealed the presence of two spectral breaks in the GBM band, $E_{\rm break}$ and $E_{\rm peak}$, coherently becoming softer from 7.5 s up to 19.5 s. Also, in this case, the spectral indices indicate synchrotron emission in the marginally fast-cooling regime. From 19.5 s onwards (approximately when the softer and dimmer emission episode starts), the low-energy break $E_{\rm break}$ is continuously approaching the lower limit of the GBM band (8 keV), presumably crossing it to enter the X-ray regime. Unfortunately, the lack of simultaneous observations in X-rays with another telescope, e.g. *Swift*/XRT, prevents us from fully tracing the evolution of the spectral break down to X-rays at later times, as was done for GRB 211211A, although see also[6].

The time-averaged *Fermi*/GBM spectrum of GRB 230307A across the T_{90} interval is best fit with a cutoff power-law with $\alpha = 1.07 \pm 0.01$ and cutoff energy 936 ± 3 keV[3]. From this, we calculate a hardness ratio (the ratio of the 50 - 300 keV photon flux to the 10 - 50 keV photon flux) of $0.88^{+0.01}_{-0.02}$. This is higher than the value for 211211A (0.57) but comfortably within the 1σ distribution of hardness ratios for canonical long GRBs (i.e. with $T_{90} > 2$ s) in the *Fermi* catalogue, which we calculate to be $0.66^{+0.51}_{-0.29}$ from the data in[7]. Like GRB 211211A before it, GRB 230307A appears to have 'typical' long GRB properties in terms of its time-averaged hardness ratio and its T_{90}. This strengthens the case for a significant number[2,8] of long-duration GRBs having been mistakenly identified as stellar collapse events.

However, in some ways, GRB 230307A differs significantly from several of the other brightest GRBs. For example, the afterglow was relatively faint, while the burst was very bright. In Supplementary Figure 3, we plot the prompt fluence in the 15-150 keV band against the X-ray afterglow brightness at 11 hours (updated from[9,10]). The general trend between the afterglow brightness and fluence is seen; the best-fit slope to this relation is approximately one. So, while there is substantial scatter, there is a direct proportionality between the fluence and the afterglow brightness. Notably, while the afterglow and prompt emission of GRB 221009A were exceptionally bright (after correcting for the heavy foreground extinction), they were in keeping with this relatively broad relationship. GRB 230307A is different. Here we extrapolate the X-ray flux to 11 hours based on the measured X-ray flux at ~ 1 day and the decay slope. We also re-calculate the GRB 230307A fluence in the relevant 15-150 keV energy band for comparison to *Swift*/BAT. This burst is a notable outlier in the relation, with a faint X-ray flux for its extraordinary prompt brightness. The afterglow brightness depends both on the energy of the burst and the density of the interstellar medium; it is, therefore, possible that the location in this fluence – afterglow brightness plane is indicative of a low-density medium, which would be consistent with expectations for such a large GRB - host offset.

It is also of interest that another burst in a similar location is GRB 211211A. This long burst has a clear signature of kilonova emission within its light curve. If GRB 230307A is a similar event, faint afterglows (relative to the prompt emission) may be an effective route for disentangling mergers from collapsars.

To further compare the ratio between the X-ray brightness and the γ-ray fluence, we retrieve the X-ray light curve of all *Swift*-detected GRBs from the *Swift* Burst Analyser[11] and limit the sample to 985 long GRBs and 55 short GRBs with at least two XRT detections and measured BAT fluence. The fluences are taken from the *Swift*/BAT Gamma-Ray Burst Catalog[1][12] and represent the measurements from 15 to 150 keV integrated over the total burst duration. We add to this sample the GRBs 170817A (off axis[13]) and 221009A (brightest GRB detected to date[1]). For the former, we retrieve the X-ray light curve from[14] and use a γ-ray fluence of 2.4×10^{-7} erg cm^{-2}[13]. For the latter, we take the X-ray light curve from the *Swift* Burst Analyser and assume a fluence of 0.007 erg cm^{-2} (corrected from the 1-10000 keV fluence in[1] to the 15-150 keV band). Following[15], we resample the X-ray light curves and normalise them by the γ-ray fluence on a grid defined by the observed F_X/Fluence ratios and the time-span probed by the data. If no data are available at a specific time of the grid, we linearly interpolate between adjacent observations but do not extrapolate any data. Hence the paucity of observations at later times reflects the last time at which sources were detected by the *Swift*/XRT.

Short and long GRBs occupy the same part of the F_X/Fluence vs time parameter space (Figure 1, main article). In contrast, GRB 230307A has an unprecedentedly low F_X/Fluence ratio that is almost 10-fold lower than the faintest GRBs at the same time. To emphasise the uniqueness of GRB 230307A, we also show in the same figure the *Swift*/BAT-detected GRBs 050925, 051105A, 070209, 070810B, 100628A, 130313A, 170112A that evaded detection with *Swift*/XRT. The limits on their F_X/Fluence ratio (shown by downward pointing triangles in that figure) are consistent with the observed range of F_X/Fluence ratios, ruling out a selection bias against GRBs with lower than usual F_X/Fluence. Intriguingly, GRBs 080503, 191019A and 211211A had markedly low F_X/Fluence ratios during the shallow decline phase of their X-ray light curves. Furthermore, GRB 211211A reached a value of 1.2×10^{-9} s^{-1} at 120 ks, comparable to GRB 230307A.

SI.1.2 Counterpart Evolution

Although the afterglow of GRB 230307A was promptly detected thanks to TESS, this data was not available to the community for several days. Further follow-up was, therefore, much slower, and the counterpart was not discovered until the localisation was narrowed down to several sq. arcminutes, approximately 24 hours after the burst. The result is that the counterpart is poorly sampled (particularly in colour) during the early phases, while later observations suffer from typically modest signal-to-noise.

The TESS observations detected a relatively bright (though not exceptional given the fluence of the burst) outburst, coincident with the prompt emission, likely peaking at $I < 15$[16]. The afterglow was much fainter, apparently no brighter than $I = 18$ in the minutes to hours after the burst was detected. It was relatively flat during this period, with a power-law through the first to last TESS observations decaying as $F(t) \propto t^{-0.2}$. The TESS and ground-based observations can be consistently modelled with a forward shock afterglow + kilonova (see Section SI.3.1).

There are no simultaneous colours at the time of the first ground-based afterglow detections (1.4 days), although extrapolation of the r-band detection with ULTRACAM to the WHITE detection with the *Swift*/UVOT suggests a relatively red colour (WHITE-r = 1.6 $\pm$ 0.4) . However, such an interpretation is difficult due both to the large photometric errors and the width of the WHITE filter on the *Swift*/UVOT.

Optical observations obtained multiple colours at an epoch ~ 2.4 days post-burst. These show the afterglow to have a blue colour with $g = 22.35 \pm 0.26$, $i = 21.68 \pm 0.09$ and $z = 21.8$[17]. This is consistent with GRB afterglows in general(i.e $F_\nu \propto \nu^{-\beta}$ gives $\beta \approx 1$). Observations in the near infrared (NIR) were not undertaken until $\sim$ 10.4 days post-burst. However, these reveal a relatively bright K-band source. The inferred i-K(AB) > 2.9 at this epoch is very red. Interpreted as a change in the spectral slope, it is $\beta \approx 2.5$. The K-band light hence appears to be in significant excess with respect to the afterglow expectations based both on optical data and on the X-ray light curve.

It is relevant to consider if such an excess could arise via extinction. However, this is not straightforward to explain. For a generic $\beta = 1$ slope we expect $i - K(AB) \approx 1.1$. At $z = 0$, to obtain $i - K(AB) = 2.9$ would correspond to a foreground extinction of $A_V \approx 4$. However, this would also predict $g - i \approx 3$, which is entirely inconsistent with the earlier observations. This problem becomes more acute for higher redshifts, where the bluer bands probe increasingly into the UV.

The IR excess becomes extremely prominent by the time of the *JWST* observations. At 28.5 days, the source is detected in all bands but is very faint in the NIRCam blue channel (F070W, F115W, F150W) and rises rapidly (in F_ν) through the redder bands (F277W, F356W, F444W). Expressed as a power-law, this is $\beta \approx 3.1$ in the 2-5 micron region, and $\beta \approx 1$ between 0.7-1.5 microns. This does not match the expectations for any plausible spectral break in a GRB afterglow or any plausible extinction (where one would expect the slope to steepen towards the blue). This strongly implies that the red excess seen in the K-band at ten days and with JWST at 28.5 days is some additional component. Indeed, in the JWST observations, the other component, beginning at around 2 microns, is very clearly visible in both photometry and spectroscopy.

This component evolves exceptionally rapidly. In the K-band, the inferred decay rate from 11.5 to 28.5 days is $\sim t^{-3.5}$ expressed as a power-law or $\sim$ 0.25 mag per day, if exponential. This is much faster than observed in GRB afterglows or supernovae. It is, however, consistent with the expectations for kilonovae. As shown in Figure 4 (main article), the overall

[1] https://swift.gsfc.nasa.gov/results/batgrbcat/index_tables.html

evolution shows substantial similarity with AT2017gfo. To constrain the temporal and spectral evolution within a plausible physical model more accurately, we fit the multi-band photometry with afterglow and kilonova models. The outputs of these models are described in detail in section SI.3.

SI.1.3 Identification of the host galaxy

Deep optical imaging of the field identifies several relatively bright galaxies in the vicinity of the sky position of GRB 230307A. Our preferred host galaxy is the brightest of these, which we denote as G1. It lies at $z = 0.065$ and is offset 30 arcseconds (40 kpc in projection) from the location of the afterglow. Following the method of[18] this galaxy has a probability of chance alignment of $P_{\rm chance} \sim 0.09$ (see also[17]). Although this is not extremely low, and so is only suggestive of a connection to the transient, we note that i) the luminosity of the late time counterpart at this redshift is very similar to AT2017gfo and ii) the spectral feature seen at 2.1 microns in AT2017gfo matches with the emission feature seen in the JWST spectroscopy of GRB 230307A. This is a broad line, but assuming they have the same physical origin, they fix the redshift to the range $0.04 < z < 0.08$. G1 is the only galaxy within this range in the field. The physical properties of this galaxy are outlined in section SI.1.4.

Our MUSE observations provide redshifts for this galaxy and several others, also identifying a small group of galaxies (G2, G3, G4) at a common redshift of $z = 0.263$. All of these galaxies have $P_{\rm chance}$ values substantially greater than our preferred host. Furthermore, because of the larger redshifts, the implied offsets from GRB 230307A are $\gg$ 100kpc. This is larger than seen for any short GRB with a firmly identified host. We, therefore, disfavour these as plausible host galaxies for GRB 230307A.

Deep JWST observations reveal no evidence of a directly underlying host galaxy for GRB 230307A, as would be expected if it had a collapsar origin. In particular, at late times, the faint source at the counterpart's location is consistent with a point-source (i.e. a subtraction of the PSF constructed by WebbPSF yields no significant residuals). However, we identify a faint galaxy, undetected in the blue and with F277W $=27.9 \pm 0.1$, offset only 0.3 arcseconds from the burst position. We designate this galaxy H1.

Our NIRSpec observations provide a redshift of $z = 3.87$ for H1 based on the detection of [O III] (5007) and Hα. At this redshift, the offset is only ~ 1.3 kpc. Although many $z \sim 4$ galaxies are extremely compact[19], it seems likely that some stellar population from this galaxy does extend under the burst position, and there may be marginal evidence for extension in this direction in the F444W image. However, this region is neither UV-bright nor an emission line region where one may expect to observe massive stars.

The galaxy photometry, performed in 0.1-arcsecond apertures and subsequently corrected for encircled energy assuming point-source curves is F070W>29.0, F115W=28.4 $\pm$ 0.3, F150W=28.6 $\pm$ 0.4, F277W=27.9 $\pm$ 0.1, F444W=28.3 $\pm$ 0.1, and the galaxy is only robustly detected in the redder bands (see Figure 2, main article). We note that because of the proximity of the afterglow, we use a smaller aperture than may be optimal, although the galaxy is also compact.

We can estimate the probability of chance alignment of this source with the GRB position via various routes. In principle, one can use number counts of galaxies on the sky in the multiple bands. These have recently been updated based on the first observations with *JWST* to provide number counts in appropriate bands[20]. We find that $P_{\rm chance}$, following the approach of[18] to be in the range $\sim$ 3-6 % for F277W and F444W (with no bound in the filters where the galaxy is undetected). Alternatively, we also estimate the probability directly from the data. We extract sources within the field via Source Extractor to create a mask of objects within the field. In the brightest detection (F277W) approximately 5% of the image is covered with objects of equal or brighter magnitude to H1, and we note that the burst position is *not contained* within this mask. This suggests that in this particular field, $P_{\rm chance} > 5\%$.

The absolute magnitude of H1 is $M_i \sim -17.7$, and the Hα star formation rate is approximately 1 M$_\odot$ yr^{-1}. The half-light radius of the galaxy is approximately 0.1 arcseconds (700 pc). Although limited information is available, these values are generally consistent with those of the long GRB population. The burst offset from its host galaxy is ~ 2.5 half-light radii. This is large but within the range seen for long-GRBs[21].

In our X-shooter and MUSE observations there is no trace visible in 1D or 2D extractions at the source position, although a weak continuum is seen in the X-shooter spectrum when heavily binned. This is consistent with its faint magnitude at the time of the observations. At the location of Lyα at $z = 3.87$ we place limits of $F < 2.5 \times 10^{-17}$ erg s^{-1} cm^{-2} assuming an unresolved line.

We also examined both spectra for any emission lines at other redshifts. This is worthwhile given the strong emission lines often seen in long GRB hosts[22], which may make emission line redshifts possible, even if the host itself is undetected. However, despite deep observations, there are no visible emission lines consistent with no directly underlying host galaxy, consistent with a compact object merger, but not a collapsar.

Unsurprisingly, there are also numerous faint galaxies in the JWST images. However, all of these have large $P_{\rm chance}$ values, and we do not consider them plausible host galaxies.

Taken a face value, the probability of chance alignment for G1 (our preferred host) and H1 ($z = 3.87$) is similar. However, the luminosity, lightcurve evolution and spectroscopic feature at the redshift of G1 offer strong support for it as the host galaxy of GRB 230307A. Furthermore, there is no straightforward, reasonably viable physical model that could explain the burst's extreme properties at $z = 3.87$. This scenario would require extreme energetics, exceptionally rapid evolution and yields unphysical outcomes in standard GRB or supernovae scenarios. We outline this in detail in section SI.4.1.

SI.1.4 Host galaxy properties

To better understand the properties of G1, the likely GRB host galaxy, we performed a fit to both the MUSE spectrum and photometric measurements from the far-UV to the mid-IR. For the photometric measurements, we retrieved science-ready coadded images from the *Galaxy Evolution Explorer* (GALEX) general release 6/7[23], DESI Legacy Imaging Surveys (LS;[24]) data release 9, and re-processed WISE images[25] from the unWISE archive[26][2]. The unWISE images are based on the public WISE data and include images from the ongoing NEOWISE-Reactivation mission R7[27,28]. We measured the brightness of the galaxy G1 using the Lambda Adaptive Multi-Band Deblending Algorithm in R (*LAMBDAR*[29]) and the methods described in[30]. We augment the SED with *Swift*/UVOT photometry in the *u* band and our 6-band JWST/NIRCAM photometry. The photometry on the UVOT images was done with *uvotsource* in *HEASoft* and an aperture encircling the entire galaxy. For JWST photometry, we used a 6-arcsec circular aperture, which allows us to gather all the observed light observed in JWST filters from the host galaxy. All measurements are summarised in Supplementary Table 2.

To derive the main physical properties of the host galaxy, such as its stellar mass, we employ two separate methodologies based on the photometric and spectroscopic data available for the host, and finally compare the results to assess the robustness of our conclusions. We first fit the multi-wavelength (0.1–4.4 μm) dataset using the `prospector` python package[31], which allows us to model the host galaxy spectrum starting from its main constituents, namely a set of stellar population base spectra, built from the Flexible Stellar Population Synthesis (FSPS) package[32], and combined with a specific star-formation history (SFH) model. Moreover, we have also considered a fixed attenuation model based on the Calzetti[33] attenuation curve, and an additional nebular model originating from the gas component, which is built using the `Cloudy` photo-ionization code[34], and considering the FSPS stellar population as ionising sources. We have adopted a parametric SFH model, which is described by a delayed-exponential model where the star-formation rate varies as a function of time $t = t_{\rm age} - t_{\rm lt}$, with $t_{\rm lt}$ being the lookback time[31], as SFR $\propto (t/\tau)\exp(-t/\tau)$, with τ being the *e*-folding time. We finally have used the `dynesty`[35] ensemble sampler to reconstruct the posterior distribution.

The results of the prospector analysis are shown in Fig. 4. We obtain a mass value of the living stars of $M_* = 2.37(+0.24, -0.35) \times 10^9$ M$_\odot$. The mass of all stars ever formed is $0.20(+0.02, -0.04)$ dex larger. The light-weighted stellar age resulting from the fit is $1.13(+1.49, -0.36)$ Gyr.

An alternative to parametric SED fits is to use synthetic stellar population SEDs as templates and combine them to fit the galactic spectra (the underlying assumption being instantaneous star formations rather than continuous functions of time). We can use the spectral synthesis from the BPASSv2.2.2[36,37] binary populations and create templates with `hoki`[38] that are compatible with the `ppxf` fitting package[39], as described in[40]. Because SED fitting has a high level of degeneracies (see[39]), at first we do not fit all 13 BPASS metallicities at once with `ppxf`, as this can result in unphysical results (see discussion in[40]); instead we fit the metallicities individually to find which ones result in the best fits on their own. We find that a low Z (0.001) population and solar metallicity population (Z=0.014) result in decent fits, but the low metallicity population fails to predict a young stellar component that is seen in the images, whilst the solar metallicity fit fails to accurately match the Hβ and neighbouring absorption features in the blue part of the spectrum. So we then fit the galaxy simultaneously with Z=0.001 and Z=0.014 templates, and retrieve a good fit shown in Figure 5 alongside the recovered SFH.

We find evidence of three main stellar populations: >95% of the mass is found in lower metallicity (Z=0.001) stars with ages ranging from a few Gyr to 10 Gyr, with a peak of star formation around 5 Gyr; >4.7% of the mass originates from a solar metallicity population (Z=0.014) that formed around 400 Myr ago; finally a small fraction (<0.05%) of the stellar mass in the host originates from the star-forming regions with ages a few Myrs.

The details of the age distributions and exact metallicity values can be model dependent so we also fit the integrated galaxy spectrum with the single stellar population synthesis code STARLIGHT[41], which uses stellar populations based on 25 different ages and six metallicity values[42], and a Chabrier IMF[43]. The SFH retrieved by this method is more complex and would require odd configurations (including some high metallicties at old ages and low metallicities around 100 Myr, which is counter-intuitive, unless inflow from pristine gas will trigger a burst of SFR), but it also finds that overall the galaxy is dominated by an old population with lower metallicity and has a younger component at higher metallicity. In Supplementary Figure 5 we show a comparison of the STARLIGHT and BPASS fits in the bottom left panel and see that they are very similar, despite STARLIGHT containing 6 different metallicities and assuming solely single star populations. This highlights the level of degeneracy we face when performing galaxy SED fits. We leave further comparisons to a follow-up study dedicated to

[2] http://unwise.me

the host and the progenitor populations of GRB 230307A, where we will also present detailed, specially resolved, fits to the datacube including its kinematics.

For now we use the BPASS integrated fits to infer the stellar mass and the star formation rate of the host of GRB 230307A, as the fit and SFH is more convincing that the one obtained with STARLIGHT. We find that there are currently $M_* = 1.65 \times 10^9 M_\odot$ of living stars (corresponding to 3.1 $\times 10^9 M_\odot$ at ZAMS) in G1. Using the nebular component retrieved from subtracting the fit of the stellar component to the observed data, we can also estimate the star formation rate and metallicity. From the Hα feature we estimate that the SFR is $5.47 \pm 0.30 \times 10^{-1}\, M_\odot \mathrm{yr}^{-1}$ using the Kennicutt formulation[44], and using the N2 index, in the CALIFA formulation[45], we infer an oxygen abundance of 8.20 $\pm$ 0.16 (12 + log(O/H)).

There are qualitative similarities between the host of GRB 230307A and NGC 4993[40], the host of the first confirmed kilonova (they are both dominated by an older stellar populations and include a younger more metal rich component), but there are some key differences: NGC 4993 was a lenticular galaxy without a clear young component, whereas the host of GRB 230307A shows clear spiral arms and star forming regions. Another major difference is that the metallicity of the old population in this galaxy is 10 times lower than that of NGC 4993 (Z=0.001 compared to Z=0.010), which will influence the stellar evolution of potential progenitors. Finally, NGC 4993 had a large stellar (and presumably dark halo) mass $M_* \approx 10^{11}$ $M_\odot$[40], a factor of > 50 larger than the host of GRB 230307A.

The location of GRB 230307A relative to its host galaxy is consistent with these properties. In particular, the low mass of the galaxy suggests a modest gravitational potential such that binaries with velocities of a few hundred km s^{-1} can readily escape. The large offset also suggests that the binary is formed from the older stellar population.

SI.1.5 Properties of the brightest GRBs

GRB 230307A is the second brightest[3] burst observed in over 50 years of observations[1]. If it arises from a compact object merger, this implies that such bright bursts can be created in mergers. Indeed, such a picture appears likely based on GRB 211211A[2,8,46], the sixth brightest burst. Of the ten brightest bursts observed by the *Fermi*/GBM, and subsequently localised at the arcsecond level, three have apparently secure associations with supernovae (GRB 130427A, GRB 171010A, GRB 190114C), and two (GRB 211211A, GRB 230307A) are associated with kilonovae, and hence mergers. Of the remaining five, one lies at $z = 1.4$ and has energetics which suggest a collapsar; three have no redshift information, although one of these (GRB 160821A) lies in proximity to several galaxies at $z = 0.19$; and one is GRB 221009A whose associated with a supernova remains unclear[47–50], although recent observations suggest a collapsar with an associated supernova is most likely[51]. Within this very bright population, collapsars are likely as common as mergers, within the substantial poisson uncertainties.

SI.2 Event rates

One key question of interest is the likely event rate for such merger GRBs. A simple estimate of the event rate associated to a single event is given by

$$R = \frac{1}{\Omega t V_{\max}}. \tag{1}$$

Here Ω reflects the fraction of the sky covered by the detection mission, t the effective mission duration (accounting for the duty cycle) and $V_{\max}$ the maximum co-moving volume within which a burst with the same properties could be identified.

For GRB 230307A, $\Omega = 0.65$ (average for the *Fermi*/GBM) and $t \approx 15$ years. $V_{\max}$ is more complicated: as shown in Supplementary Figure 2, the fluence distribution for GBM bursts extends to $\sim 10^{-8}$ erg cm^{-2} and is likely complete to around 10^{-6} erg cm^{-2}. Given the extreme brightness of GRB 230307A, it would likely have been recovered to a distance ~ 50 times greater than its observed distance. If at $z = 0.065$ the inferred $z_{\max} = 2.03$ or $V_{\max} = 630$ Gpc^{-3}. In this case, the inferred rate of such bursts becomes extremely small, $R \approx 1.6 \times 10^{-4}$ Gpc^{-3} yr^{-1}. However, in practice, such bursts would not readily be identified at such redshifts since neither supernova nor kilonova signatures could be observed. A more realistic estimate would correspond to the distance at which associated supernovae can be either identified or ruled out with moderate confidence. In this case $z_{\max} = 0.5$ (also adopted by[52]), $V_{\max} = 29$ Gpc^3, and $R \approx 3.5 \times 10^{-3}$ Gpc^{-3} yr^{-1}.

These rate estimates also assume that GRB 230307A is the *only* merger-GRB to have occurred within the 15-year lifetime of the *Fermi*/GBM. This is almost certainly not the case. Indeed, GRB 211211A was also identified by *Fermi*/GBM and has rather similar estimates of the intrinisc rate (5.7×10^{-3} Gpc^{-3} yr^{-1},?).

However, even the interpretation of ~ 2 events is problematic. In particular, the $V/V_{\max}$ for GRB 230307A is 0.004, and for GRB 211211A = 0.005 (again assuming $z_{\max} = 0.5$). For a sample average of uniformly distributed sources of comparable energy or luminosity, we expect $V/V_{\max} \sim 0.5$. That the initial identification of such a population should arise from bursts with such extreme $V/V_{\max}$ values is surprising, but may reflect that these bursts are the brightest, which likely encouraged a detailed

[3]We use brightest here as an indicator of the total fluence in the prompt emission

follow-up. However, it is improbable that they represent the only such bursts observed, and we should expect a much larger population.

To better quantify this, we extend our analysis to the *Swift* bursts and utilise the fluence of GRB 230307A converted to a 15–150 keV equivalent fluence using the observed spectral parameters. At $z = 0.065$, $E_{\rm iso}$(15–150) keV $\sim 7 \times 10^{51}$ erg, and for GRB 211211A $E_{\rm iso}$(15-150) keV $= 2 \times 10^{51}$ erg. As expected, low energy events dominate the low redshift GRB population. However, at $z < 0.5$, there are 12 (out of 42) bursts with $E_{\rm iso} \gtrsim 10^{51}$ erg. This includes some further supernova-less GRBs, in particular GRB 060614 ($E_{\rm iso} = 9 \times 10^{50}$ erg, where a KN has been suggested[53,54]), GRB 191019A ($E_{\rm iso} = 2.0 \times 10^{51}$ erg), and some bursts for which supernova searches have not been reported (e.g. GRB 150727A, GRB 061021, and the 'ultra-long' GRB 130925A). This sets an upper limit on the number of bursts at low redshift, which may be associated with mergers. In practice, selection effects would support a scenario where mergers generate a larger fraction of these bursts. In particular, the afterglows of GRB 230307A and GRB 211211A appear to be faint, despite the bright prompt emission. Such afterglows are difficult to find and may evade detection. In these cases redshifts may only be obtained from host galaxies. The associations may not be obvious if the bursts are offset from host galaxies at moderate redshifts. Such follow-up may occur late after the burst, or optical afterglow non-detections may lead to a lack of optical/IR follow-up because of uncertainty regarding the optical brightness of the event or suggestions it may be optically dark because of host galaxy extinction. Finally, given the afterglow brightness issues, it is possible that the small fraction of bright GRBs without redshift measurements may arise from a similar channel. These observations would imply that between 30-70% bursts at $z < 0.5$ and $E_{\rm iso} \gtrsim 10^{51}$ erg could arise from mergers, although it is likely less. A modest number of events at higher redshift is consistent with the observations, and would alleviate concerns regarding V/V_{max} for GRB 211211A and GRB 230307A.

This fraction is surprisingly high given the strong evidence that long GRBs arise from broad-lined type Ic supernovae and short GRBs from compact object mergers. However, the dominant contributors to the long-GRB supernova connection occur at low energy, and belong to a population of low luminosity GRBs (LLGRBs)[55]. In a significant number of these, we may observe a energy source in the prompt emission separate from the highly relativistic jet seen in on-axis, energetic bursts. For example, the long-lived, soft nature of some bursts suggests a contribution from shock breakout or cocoon emission. If, for this reason, the luminosity function of collapsar GRBs is steeper at low luminosity than that of merger-GRBs, it is possible that at low luminosity the long GRB population is dominated by collapsars, while at high luminosity the contribution of mergers is significant. Such an interpretation is not without problems, given the star-forming nature of long-GRB hosts and their typically small offsets from their host galaxies. However, it is a logical investigation for future work.

SI.3 Modelling

SI.3.1 Light curve modelling

In order to shed light onto the properties of the jet and, even more importantly, to separate the contribution of the kilonova from that of the jet afterglow in the UVOIR bands, we modelled the multi-wavelength light curves from radio to X-rays as a superposition of synchrotron emission from the forward shock driven by the jet into the interstellar medium (ISM), following[56,57], and blackbody emission from the photophere of a kilonova, using the simple single-component model of[58].

The forward shock synchrotron emission model has eight parameters, namely the isotropic-equivalent kinetic energy in the jet $E_{\rm K}$, its initial bulk Lorentz factor Γ_0, its half-opening angle $\theta_{\rm j}$, the ISM number density n, the fraction $\xi_{\rm N}$ of ISM electrons that undergo diffusive shock acceleration in the forward shock, the fraction $\varepsilon_{\rm e}$ of the shock downstream internal energy that is shared by such electrons, the slope p of the power law ${\rm d}N_{\rm e}/{\rm d}\gamma \propto \gamma^{-p}$ that describes the Lorentz factor (as measured in the shock downstream comoving frame) distribution of the accelerated electrons as they leave the acceleration region, and the fraction $\varepsilon_{\rm B}$ of the shock downstream internal energy that is shared by a small-scale, turbulence-driven, random magnetic field. The shock hydrodynamics is computed from energy conservation and accounts for the lateral expansion of the shock[56]. The effective electron energy distribution is computed accounting for the cooling induced by synchrotron and synchrotron-self-Compton emission, including an approximate treatment of the Klein-Nishina suppression of the Thomson cross section[56]. In computing flux densities, the synchrotron surface brightness of the shock is integrated over equal-arrival-time surfaces to account for the effects of relativistic aberration and latitude-dependent retarded times on the spectral shape[59].

The kilonova model[58] assumes spherical ejecta expanding homologously, $v = r/t$, and featuring a power law density profile $\rho(r,t) \propto t^{-3} v^{-\delta}$ between a minimum and a maximum velocity, $v_{\rm ej} \leq v \leq v_{\rm ej,max}$. The density normalization is set by the total ejecta mass $M_{\rm ej}$. In general, the model allows for the ejecta opacity (assumed grey) κ to be piecewise-constant within the profile, but here we assume a uniform opacity across the ejecta for simplicity. The model divides the ejecta into 100 small shells and computes the heating rate and thermalization efficiency within each. This allows for the derivation of the internal energy evolution in each shell and eventually the computation of the photospheric luminosity $L_{\rm KN}$ in the diffusion approximation. The fixed ejecta opacity also allows for the computation of the optical depth and hence for the identification of a photospheric radius, which then sets the effective temperature $T_{\rm KN}$ by the Stefan-Boltzmann law. In our modelling of GRB 230307A, we

computed the flux density by simply assuming pure blackbody emission with the given luminosity and effective temperature at each given time. We fixed $v_{\rm max} = 0.6c$ and left $M_{\rm ej}$, $v_{\rm ej}$, κ and δ as free parameters.

To carry out the model fitting, we defined an asymmetric Gaussian log-likelihood term for the i-th datapoint, which corresponds to an observation at time t_i and in a band whose central frequency is ν_i, as

$$\ln \mathscr{L}_i = -\frac{1}{2}\frac{(F_{\nu,\rm m}(\nu_i,t_i) - F_{\nu,\rm obs,i})^2}{\sigma_i^2 + f_{\rm sys}^2 F_{\nu,\rm m}^2} - \ln\left[\sqrt{2\pi\left(\sigma_{{\rm l},i}^2 + f_{\rm sys}^2 F_{\nu,\rm m}^2\right)} + \sqrt{2\pi\left(\sigma_{{\rm h},i}^2 + f_{\rm sys}^2 F_{\nu,\rm m}^2\right)}\right], \tag{2}$$

where $F_{\nu,\rm m}(\nu,t)$ is the flux density predicted by the model, $F_{\nu,{\rm obs},i}$ is the measured flux density, the one-sigma error reflects the potentially asymmetric error bars

$$\sigma_i = \begin{cases} \sigma_{{\rm l},i} & \text{if}\, F_{\nu,\rm m}(\nu_i,t_i) \leq F_{\nu,{\rm obs},i} \\ \sigma_{{\rm h},i} & \text{if}\, F_{\nu,\rm m}(\nu_i,t_i) > F_{\nu,{\rm obs},i} \end{cases}, \tag{3}$$

and we introduced a fractional systematic error contribution $f_{\rm sys}$, which we take as an additional nuisance parameter, to account for potential inter-calibration uncertainties between different instruments and for the fact that error bars typically only account for statistical uncertainties. For X-ray detections, we fit the integrated flux and the spectral index independently, with an analogous term for each (but with no systematic error contribution for the spectral index). Upper limits were treated simply by setting $F_{\nu,{\rm obs},i}$ equal to the reported upper limit, $\sigma_{{\rm h},i} = F_{\nu,{\rm obs},i}/10$ and $\sigma_{{\rm l},i} = 10F_{\nu,{\rm obs},i}$. The final log-likelihood was taken as the sum of these terms.

In order to derive a posterior probability density on our 13-dimensional parameter space, we assumed the priors reported in Table 3 and we sampled the posterior with a Markov Chain Monte Carlo approach using the `emcee` python module[60], which implements the Goodman and Weare[61] affine-invariant ensemble sampler. The medians and 90% credible intervals of the marginalised posteriors on each parameter obtained in this way are reported in Table 3. The posterior is visualised by means of corner plots in Supplementary Figures 6 (jet afterglow parameters), 7 (kilonova parameters) and 8 (all parameters).

The left-hand panel in Supplementary Figure 9 shows the observed light curve data (markers) along with the best-fitting model (solid lines). Dashed lines single out the contribution of the kilonova. The right-hand panel in the same figure shows some selected spectra, showing in particular the good agreement of the first JWST epoch with the blackbody plus power law spectrum implied by our model at those times.

While the best-fit model demonstrates a relatively good agreement with most of the measurements, some discrepancies stand out, most prominently with the 61.5 d JWST data and with the 28.5 d *Chandra* detection. The former is not too surprising, as the assumptions in the kilonova model (in particular that of blackbody photopsheric emission, which is particularly rough in such a nebular phase, and that of constant and uniform grey opacity, due to recombination of at least some species) are expected to break down at such late epochs. The latter is linked to the steepening ('jet break') apparent at around 2 days in the model X-ray light curve, which in turn is mainly driven by the need to not exceed the optical and near-infrared fluxes implied by observations at around one week and beyond. In absence of these constraints, the fit would have accommodated a larger jet half-opening angle, postponing the jet break and hence allowing for a better match with the best-fit *Chandra* flux. On the other hand, as noted in Methods, this flux is rather uncertain, with the low-end uncertainty possibly extending to fluxes lower by one order of magnitude or more, depending on the adopted prior in the spectral analysis (see Methods). Still, such a discrepancy might indicate the presence of additional X-ray emission that is not accounted for by the model, as has been seen previously in e.g.[62,63].

SI.3.2 Spectral analysis modeling

The JWST/NIRSpec spectrum taken on 5 April 2023 exhibits a red continuum component with emission line features. The most distinctive feature is a broad emission line at 2.15 microns (in the rest frame, assuming $z = 0.065$). This may be a blend (visibly split in Figure 3, main article) and a simultaneous fit of two Gaussians provides measured centroids of 20285±10Å and 22062±10Å. The line widths are both consistent at $v_{\rm FWHM} = 19100\,{\rm kms}^{-1}$ (0.064c). This 2.1 micron feature is quite similar in strength and width to the 2.07 micron feature in AT2017gfo at 10.5 days after merger (discussed in[64]). The AT2017gfo line also appears to be better fit as a blend of two features rather than a single transition, with line velocities of $v_{\rm FWHM} = 38900\,{\rm kms}^{-1}$. While the average line centre is reasonably consistent between the two, the components inferred for AT2017gfo and the kilonova of GRB 230307A are each quite different. Reference[64] finds them at 20590Å and 21350Å and there is no consistent velocity shift that could be applied to match AT2017gfo with our JWST spectrum. Nevertheless, the similarity in their average line centroids, velocities and equivalent widths is striking, as demonstrated in Figure 3 (main article).

With a Doppler broadening parameter of $\lesssim 0.1c$, it is unlikely that the continuum component is formed as a result of the superposition of emission lines. Because kilonova radiation transfer at such late times is not yet fully understood, here we attempt to model the spectrum with the assumption that the emission consists of blackbody radiation from the photosphere and forbidden emission lines of heavy elements formed outside the photosphere.

If the continuum is described with blackbody radiation, the temperature and photospheric velocity are $\approx 670\,\mathrm{K}$ and $\approx 0.08c$, respectively. The continuum luminosity is estimated as $\sim 2\times10^{39}\,\mathrm{erg/s}$ in the NIRSpec band and $\sim 5\times10^{39}\,\mathrm{erg/s}$ if the blackbody emission extends to much longer wavelengths. Assuming this emission is entirely powered by radioactivity of r-process nuclei, these correspond to an ejecta mass of ~ 0.03–$0.07M_\odot$[58]. With the ejecta mass and velocity, the opacity is required to be $\gtrsim 5\,\mathrm{cm^2/g}$ in order to keep the ejecta optically thick at 30 day. It is worth noting that such a high opacity in the mid-IR indicates that the inner part of the ejecta is lanthanide rich[65–67]. Another scenario for the high opacity at low temperatures is that dust grains of heavy elements exist in the ejecta. A previous study has shown that the dust formation in kilonova ejecta is unlikely to occur[68]. However, the ejecta density at dust formation used in the study is somewhat lower than the one inferred from our analysis for GRB 230307A. Thus, the dust formation might occur in the inner part of the ejecta.

Forbidden emission lines in the infrared are expected to arise from fine structure transitions of low-lying energy levels of heavy elements. Most abundant ions are expected to produce the strongest lines. We attribute the strongest observed line at 2.15 microns to tellurium (Te) III from an M1 line list of heavy elements presented in[69], where the line wavelengths are experimentally calibrated according to the NIST database[70]. Te belongs to the second r-process peak. With the M1 line list, we model kilonova emission line spectra under the assumption that photons from forbidden lines produced outside the photosphere freely escape from the ejecta. The collision strengths of Te III are taken from an R-matrix calculation[71] and those of other ions are obtained by using an atomic structure code HULLAC[72]. The abundance pattern is chosen to be the solar r-process but we separate "light" and "heavy" elements at an atomic mass of 85 and introduce a parameter, the abundance ratio of the two (see Supplementary Figure 10). The ionization fractions are fixed to be $(Y^{+1}, Y^{+2}, Y^{+3}) = (0.2, 0.5, 0.3)$ motivated by the Te ionization evolution in kilonova ejecta[73]. The line shape is approximated by a Gaussian with a line broadening velocity of $0.08c$, which is the same as the photospheric velocity. The mass in the line forming region is estimated by assuming that the observed line luminosity, $5\times10^{38}\,\mathrm{erg\,s^{-1}}$, is locally generated by radioactivity of r-process nuclei, corresponding to $\sim 0.02M_\odot$. Given the abundance pattern and ionization state, the mass of Te III in the line forming region is $\approx 8\cdot10^{-4}M_\odot$. The electron temperature of the line forming region is then determined such that the total line luminosity agrees with the observed one. The estimated electron temperature is $\sim 3000\,\mathrm{K}$, which is slightly lower than that derived from the pure neodymium nebular modeling[74]. This is because the cooling by tellurium ions is more efficient than neodymium.

We find that the [Te III] $2.10\,\mu$m line is indeed the most outstanding emission line around 2 microns. Several weaker lines also contribute to the flux around 3–4 microns. There is another potential line feature around 4.5 microns in the NIRSpec spectrum. The location of this feature is consistent with [Se III] $4.55\,\mu$m and [W III] $4.43\,\mu$m as pointed out by[69] for the kilonova AT2017gfo. From the spectral modeling, we obtain the total ejecta mass of ~ 0.05–$0.1M_\odot$, which agrees with the one obtained from the light curve modeling $\sim 0.1M_\odot$.

Here we show a brief estimate of the Te III mass from the observed line at 2.15 microns ($^3\mathrm{P}_0$–$^3\mathrm{P}_1$). The collisional excitation rate per Te III ion from the ground level ($^3\mathrm{P}_0$) to the first exited level ($^3\mathrm{P}_1$) is given by

$$k_{01} = \frac{8.63\cdot10^{-6} n_e}{\sqrt{T_e}}\frac{\Omega_{01}}{g_0}e^{-E_{01}/kT_e}\,\mathrm{s}^{-1}, \tag{4}$$

where n_e and T_e are the thermal electron density and temperature, $\Omega_{01} \approx 5.8$ is the collision strength[71], $E_{01} \approx 0.6\,\mathrm{eV}$ is the excitation energy, g_0 is the statistical weight of the ground level. Assuming that the ejecta mass in the line forming region is $0.02M_\odot$ expanding with $0.08c$ and the ions are typically doubly ionised, we estimate $n_e \sim 3\cdot10^5\,\mathrm{cm^{-3}}$, and thus, the line emissivity per Te III ion is

$$\varepsilon_{10} \approx 2.5\cdot10^{-14}\left(\frac{n_e}{3\cdot10^5\,\mathrm{cm^{-3}}}\right)\mathrm{erg/s}, \tag{5}$$

where $T_e = 3000\,\mathrm{K}$ is used. Combining the line emissivity with the observed line luminosity in $2.25\pm0.23\,\mu$m, $L_{\mathrm{line}} \approx 3\cdot10^{38}\,\mathrm{erg/s}$, we obtain

$$M(\mathrm{Te\,III}) \approx 10^{-3}M_\odot\left(\frac{n_e}{3\cdot10^5\,\mathrm{cm^{-3}}}\right)^{-1}\left(\frac{L_{\mathrm{line}}}{3\cdot10^{38}\,\mathrm{erg/s}}\right). \tag{6}$$

The mass estimated from the line is somewhat dependent on T_e and n_e. However, we emphasise that, with $T_e \approx 3000\,\mathrm{K}$ and $n_e \approx 3\cdot10^5\mathrm{cm^{-3}}$, the line luminosity is consistent with the radioactive power in the line forming region. It is also interesting to note that the Te III mass of $10^{-3}M_\odot$ is in good agreement with the one obtained based on the same line seen in AT2017gfo at 10.5 day[75].

We should also consider the implications of the second epoch NIRSpec observations. Although contamination from the diffraction spike of the nearby star means that absolute fluxes should be treated with caution, especially in the blue, this spectrum appears to show a bluer underlying spectral slope, introduced either by the change in colour of the associated kilonova,

or the growing importance of afterglow emission. The spectral feature at 2.1 μm is apparently still present in this spectrum, and has become relatively more important compared to the continuum, with the ratio of peak flux at 2.1 microns to the 4.4 micron emission evolving from ~ 1 at 29 days to ~ 3.5 at 61 days. This supports our interpretation that the line is excited through collision with thermal electrons rather than the continuum photons. The observed flux from 1 to 5 μm decreases by a factor of ~ 4, which is consistent with the expected decline of the radioactive heating rate around the thermalization time of beta decay[58,76,77]. On the other hand, the line flux decreases only by a factor of ~ 2, indicating that the electron temperature and/or the mass in the line forming region increase from 29 to 61 days. This is consistent with a scenario in which the temperature structure is changing within the expanding ejecta.

While we conclude that the observed line feature at 2.1 microns is most likely attributed to Te III, it is important to note that there are caveats associated with our modeling and there exist other candidate ions that have lines around 2.1 μm summarized in[64]. Among the candidate elements Te and Ba are expected to be the most abundant because they belong to the second r-process peak. The radiative transition rate of [Ba II] 2.05 μm[78], however, is lower by a factor $\sim 10^2$ than that of the Te III line, which makes the Ba II line much weaker. Our modeling relies on the collision strengths computed with an R-matrix method for Te III[71] and HULLAC for other ions and on the ionization degrees predicted by[73]. However, the collisional strengths obtained with HULLAC may not be as accurate as those with the R-matrix method and recombination rates are not fully understood. Therefore, improving the atomic data might affect the identification of the line.

Another caveat is that the model does not include E1 lines. Lanthanides and actinides have E1 transitions between low-lying levels in the mid-IR. Due to their lower abundances, these lines are expected to be weaker compared to the Te line if collisional excitation dominates the excitation processes. However, as we make an implicit assumption that their E1 lines contribute to the opacity in the mid-IR, they may produce P-Cygni like features, see, e.g.,[64,79]. For example, Ce III has a strong line at 2.07 μm with $\log gf = -1.67$[79]. We estimate that its line optical depth is $\lesssim 0.1$ at 700 K with $\sim 0.05 M_\odot$ and $\sim 0.1c$ even if Ce is purely in Ce III. Furthermore, it seems difficult for this E1 interpretation to account for the large excess of the line feature relative to the continuum level in the second epoch NIRSpec observation. However, more careful analyses including non-LTE effects are needed to quantify it. Including this effect may also affect the spectral modelings.

SI.4 Alternative progenitor possibilities

Our interpretation of GRB 230307A provides a self-consistent model for the source in which the temporal and spectral evolution, as well as the source location, can be readily explained. The kilonova has marked similarities with AT2017gfo providing a robust indication of its origin, and we do not need to postulate new and unseen phenomena to explain it. However, it is also relevant to consider alternative possibilities. In particular, given the location of the galaxy at $z = 3.87$, it is important to consider if the burst could originate at that redshift.

SI.4.1 GRB 230307A as a high redshift, highly energetic GRB

The nearby galaxy H1 (F277W(AB)=27.5± 0.1 , $r_{proj} = 0.3$ arcsec) with a spectroscopic redshift of z=3.87 has a relatively low probability of occurring by chance ($\sim 5-10\%$, see section SI.1.3). This galaxy has a comparable P_{chance} to G1 (the $z = 0.065$ galaxy). The host-normalised offset for H1 is ~ 2.5, which is large but not unprecedented for long GRBs[21]. However, assuming the late time light at the GRB position is all from the transient, it does not lie on the stellar field of this galaxy, which is unusual, for example, in the samples of[21,80,81], there is only one (of > 100) sub-arcsecond localised GRB not on the stellar field of its host.

At $z = 3.87$, the inferred isotropic energy release and luminosity of GRB 230307A would be $E_{\rm iso} = 1.2 \times 10^{56}$ erg and $L_{\rm iso} = 1.7 \times 10^{56}$ erg s^{-1} (using a 64 ms peak flux). This is approximately an order of magnitude more energetic and two orders of magnitude more luminous than any other previously identified GRB[1].

If at $z = 3.87$, we can have some confidence that GRB 230307A would be the most energetic burst ever detected by *Swift* or *Fermi*, including those without redshift or even afterglow identifications. In Table 1, we tabulate the most fluent GRBs observed by *Fermi*. Most of these have either redshifts or optical detections, which constrain $z < 6$ via the detection of the source in the optical band. This leads to a set of measured or maximum $E_{\rm iso}$ values. For events without any redshift information, we can place a conservative upper redshift limit of $z = 16$. No GRBs detected by *Fermi* without a redshift can have energy over 10^{56} erg unless they lie beyond $z \sim 20$. Hence, GRB 230307A is sufficiently rare, if at $z = 3.87$, that events like it occur less frequently than once per decade across the Universe (i.e. no more than one in the combined lifetimes of *Swift* and *Fermi*).

The energetics of the burst at this redshift would lie far beyond those of the general GRB population and beyond those suggested as the upper limit for GRB energetics[82]. The only population of core-collapse GRBs whose energetics have been suggested to approach this value are those from first-generation population III stars[83–85]. It is not expected that such stars should exist at $z \sim 4$. However, while a pop-III origin may alleviate energy concerns, the properties of the GRB and its optical/IR counterpart do not resemble the predictions for pop-III stars. In particular, pop III GRBs are suggested to have particularly long

durations given the mass and radii of their progenitors[85], and so require extremely long durations of engine activity to enable jet breakout. However, the ~ 35 s duration of GRB 230307A and its rapid variability do not readily fit this expectation.

If one ascribes the GRB to a stellar collapse event, considering the afterglow's properties and associated supernovae is also relevant. Firstly, at 28.5 days, the JWST spectral observations are inconsistent synchrotron emission, suggesting that the counterpart must be dominated by another source in the mid-IR and the earlier K-band points. This excess, which in our preferred model is explained by a kilonova, would have to be due to the supernova or shock breakout if at $z = 3.87$. The K-band (rest frame B-band) light would reach a peak of $M_B(AB) < -23.5$ on a timeframe of <2-days (rest-frame) before decaying at a rate of > 1 mag day^{-1} for the next four days, or as a power-law decay, a rate of approximately t^{-4}. This appears too rapid for radioactively powered transients, at least based on the sample to date (note that at $z = 3.87$, the timescales are a factor of ~ 5 faster than in the $z = 0.065$ scenario due to cosmological time dilation). The most likely option for such emission would be shock-breakout, which may begin blue but rapidly cool. There are simulations for the shock breakout associated with pop-III supernovae, which show an early peak[86]. However, this emission peaks in the UV to soft X-ray regime and at luminosities below that seen in GRB 230307A. Indeed, taking 28.5 days as a baseline; for a plausible maximum Pop III radius (e.g. 2000 $R_\odot$,[87]) and a luminosity of $L_{bol} \sim 10^{43}$ erg s^{-1} the inferred temperature is $T \sim 30,000$ K. This is incompatible with the spectral shape seen in the counterpart to GRB 230307A which peaks at > 4.5 microns ($T < 3000$K at $z = 3.87$). Dust or metal line blanketing could alleviate this discrepancy to some degree, but it would be extreme to explain the observations. It would also come at the cost of an even higher intrinsic luminosity. Conversely, the radius at which the luminosity and temperature would be consistent is extremely large (~ 0.3 pc) and indeed would require super-luminal expansion to reach from a single explosion within the time since burst. These constraints become even more extreme for the K-band observations at 11.5 days, where the luminosity is > 50 times higher. However, we lack detailed information regarding the spectral shape at this time. We can conclude that a thermal transient launched at the time of GRB 230307A cannot explain the observed source at $z = 3.87$.

We should consider if GRB 230307A could be related to an explosion which bears little to no similarity to long-GRB progenitors. Given the inferred energetics, this is not an unreasonable proposition. However, the emission is too bright and too fast for, for example, the fast blue optical transients (e.g.[88]), the fastest of which have half-times of ~ 4 days (c.f. < 1 day for GRB 230307A at $z = 3.87$;[89]). A further alternative may be a relativistic tidal disruption event. This would face significant challenges with the rapid variability timescales seen in the prompt emission and the non-nuclearity of the source within the galaxy at $z = 3.87$. Putting aside these concerns, the peak optical/IR luminosity is comparable to AT2022cmc[90,91], but the evolution is too rapid and the dynamic range too large.

Finally, it is possible that the red excess seen at later times is not directly related to the progenitor or the transient but is a result of the re-processing of the GRB radiation by material within the host galaxy. In particular, for GRB 211211A[92] suggest that an alternative explanation for the emission could be the heating of dust. However, this model also encounters significant issues at $z = 3.87$. In particular, the observed K-band excess is a rest-frame B-band excess, much bluer than expected for dust heating. If this represented the peak of the thermal spectrum, it would be above the sublimation temperature of the dust. Alternatively, if it were the blue tail of a much cooler black body, the luminosity would be extremely high.

A final challenge to the high$-z$ scenario is that the afterglow is detected in the UVOT-white and ground-based g-bands. These observations all have substantial sensitivity blue-ward of Lyα at z=3.87. A typical column from the intergalactic medium should attenuate $\sim 50\%$ of the light in these bands, inconsistent with observations. Indeed, for a typical $\beta = 1$ spectrum, we expect to observe $white - i$ = 2.8 and $g - i$ = 1.9, approximately 3 and 4 σ away from the observed colours. There is significant variation in the absorption strength as a function of the line of sight, so a low (or near zero) absorption column would alleviate this tension. The sample of[93] implies that at $z \sim 4$, perhaps 10% of galaxies have such low absorption sight lines.

Hence, while the proximate galaxy could indicate a high redshift, there are few other indications in the transient properties that would support this interpretation. In particular, neither standard thermal nor non-thermal emission can explain the observed counterpart properties. If the burst does arise from $z = 3.87$, it requires a new kind of explosion, unlike any seen until now. In practice, such explosions could be extremely rare: the volumetric rate of GRBs with $E_{\rm iso} > 10^{56}$ erg is minimal, but postulating them is unnecessary when a robust, physically motivated explanation can be obtained for a lower redshift solution.

SI.4.2 Other cosmological scenarios

We should also consider a further option which is that GRB 230307A does not reside at either $z = 0.065$ or $z = 3.87$ but is a chance super-position with both galaxies. In this scenario, the actual host is undetected or is one of the other galaxies within the field. The absence of direct redshift measurements makes placing constraints on this scenario challenging. However, we can use the non-detection of the late-time JWST magnitudes to limit the brightness of a supernova component at any redshift.

To quantify the exclusion of "normal" long GRBs at intermediate redshift we utilize model light curves for SN 1998bw from MOSFiT[94] calculated at a range of redshift from $0.05 < z < 4.0$ (we take $z = 4$ as an upper limit for the redshift the GRB based on the observed g-band detections together with the detection of continuum emission to 5300Å[95] and similar faint trace seen to ~5100Åin our X-shooter spectrum). At each redshift, we compare our observed JWST photometry in each

band to the model predictions at that time and report the most constraining limit (e.g. the lowest ratio of F_{obs}/F_{98bw}). This is shown in Supplementary Figure 11. At all plausible redshifts, any supernova must be at least a factor ~ 3 fainter than SN 1998bw at similar times. For any redshift where the burst energetics fall within the range seen in the bulk GRB population ($z < 1.2$), any supernova must be a factor > 100 fainter than SN 1998bw. Hence, there is no route in which GRB 230307A can arise from a classical long GRB ($E_{\rm iso} < 10^{55}$) erg with an associated broad-lined Ic supernova. The strength of this rejection is predominantly based on the faintness of the source in the bluer bands, whereas at the first epoch, the redder bands are substantially brighter than SN 1998bw at $z = 3.87$.

If the burst lies at an intermediate redshift, dust models may become more appealing. In particular, for a low to moderate redshift, the luminosty and timescales may be a suitable match (e.g. for GRB 211211A[92] find plausible explanations at $z \sim 0.5$). However, in this case, the lack of a supernova to extremely deep limits would be surprising, as would the non-detection of the host galaxy.

SI.4.3 GRB 230307A as an unusual supernova

It is also relevant to consider if GRB 230307A could arise from another supernova-like event, unlike those previously associated with GRBs (of any kind). GRB production requires a central engine, and so SN Ia like events, which do not provide a central neutron star or black hole should not create GRBs.

However, there is now a large diversity in the properties of other supernovae, the majority of which arise via stellar core collapse in massive stars. In the case of GRB 230307A, the galactic location strongly disfavours a collapsar-like event at $z = 0.065$. Even accounting for binary interactions which can lead to delayed supernovae[96], there is essentially no route to obtain a massive star collapse at an offset of 40 kpc from the host galaxy. This provides the strongest evidence against a massive star progenitor for GRB 2300307A. We note that at higher redshifts, at least out to the $z = 3.87$ considered in detail above, there are no other proximate galaxies for which measured offsets would not pose equally strong constraints.

It is also relevant to consider how, if the concerns regarding the location could be overcome the lightcurves and spectral evolution of alternative progenitor models may appear.

There are now populations of faint, fast supernovae which are being uncovered in increasing numbers by synoptic sky surveys[97]. These represent a natural contaminants for kilonovae in, for example, searches of gravitational wave error boxes. However, while the peak magnitudes of such systems can match the observed magnitudes seen for GRB 230307A, and some events can even transition from blue to red[98], the evolution timescales are much longer than seen for AT2017gfo. In part, selection effects may account for this as the most rapidly evolving transients are the most difficult to identify in real time. However, there are no known optical/IR supernovae which evolve on the time required for GRB 230307A.

Secondly, and of particlar relevance for GRBs, is the possible population of GRBs created in fallback supernovae in which a weak supernova is launched but the GRB is formed via the fallback of material[99,100]. The presence of weak supernovae may be observationally selected against in samples of GRB-SNe to date because the bright, long-lived SN Ic are more readily seen. Fallback supernovae would have low nickel yields and hence peak luminosities, and because of the small ejecta mass would evolve quickly. Models in the literature find temperatures of a few thousand Kelvin at relevant timescales[100], still somewhat hotter than GRB 230307A, but these models do not explore the full plausible parameter space and cooler transients may be possible. However, such progenitors do still require a massive star, and so are not readily explained in the environment of GRB 230307A.

Hence, the combination of the difficulties in modelling the progenitor as a massive star and the location within the host where no such stars are expected leads us to strongly disfavour a $z = 0.065$ collapsar in this case.

SI.4.4 Galactic objects

In the absence of a robust absorption redshift, it is also necessary to consider if GRB 230307A could arise from a Galactic system.

The very faint magnitudes and extreme red colours observed at late times can effectively rule out X-ray binary outbursts. For example, with an M-dwarf companion (absolute magnitude 9), the distance to the source would be ~ 100 kpc, and larger for any more massive star, while the late-time colours of the source are not stellar. In practice, given that the source is transient, and we may also expect some contribution from an accretion disc, the overall properties cannot be remedied within the accreting binary framework.

The inferred energetics for Galactic systems are $E_{\rm iso} \sim 5 \times 10^{43} (d/10\,{\rm kpc})^2$. This energetic output is within the bounds of giant outbursts from magnetars. For example, the giant flare of SGR 1806-20 had an inferred isotropic energy of $E_{\rm iso} \sim 2 \times 10^{46}$ erg (see[101], although subsequent downward revisions in its distance lower this somewhat[102]). However, GRB 230307A does not appear to meet the requirements of a magnetar. In particular, it is at high Galactic latitude (-36 degrees) and far from any plausible star formation that could give rise to a young neutron star. Furthermore, the emission in all magnetar outbursts is dominated by a very short pulse followed by decaying emission in which the pulse period of the neutron star is visible. This is not the case for GRB 230307A.

Burst	fluence$\times10^{-3}$ (erg cm^{-2})	t_{90} (s)	F_X (11 hour)	z	$E_{\rm iso}$ (10^{52} erg)	Notes & Refs
221009A	21	325.8	1.1 $\times10^{-9}$	0.15	1150	SN[50,51,110]
230307A	3.2	34.6	6.0 $\times10^{-13}$	0.065/3.87	2.34/12000	This work
130427A	2.46	138.2	1.0 $\times10^{-10}$	0.34	72.3	Spectroscopic SN[111]
160625A	0.64	43.0	1.1 $\times10^{-12}$	1.41	336.2	Too high-z for SN/KN
171010A	0.63	107.3	1.5 $\times10^{-11}$	0.33	17.4	Spectroscopic SN likely[112]
160821A	0.52	43.0	-	< 6	< 1300	No SN/KN
211211A	0.50	34.3	4.7 $\times10^{-12}$	0.08	0.74	Kilonova[2,8,46]
190114C	0.43	116.4	1.1 $\times10^{-11}$	0.42	19.6	Spectroscopic SN[113]
190530A	0.37	18.43	2.5 $\times10^{-11}$	< 4.5	< 1400	No afterglow
221023A	0.34	39.2	-	< 16	< 7800	No afterglow

Supplementary Table 1. The properties of the brightest 10 bursts observed by *Fermi*/GBM. Eight of these have afterglow identifications which place limits on the redshift, while it is likely that all originate from $z < 16$. As expected for the (observationally) brightest bursts there is a preference for low redshift, with the bursts all arising at much less than the mean for *Swift* GRBs[114]. In 4 cases a supernova has been identified, meaning the bursts arise from core collapse. In 2 cases a kilonova origin appears more likely, while for the remaining 4 the redshifts (or absence thereof) render such diagnostics impossible. However, it would appear that samples of bright, long-GRBs may contain significant number of compact object mergers.

GRBs have previously been suggested to arise from the tidal disruption and accretion of rocky material onto a neutron star[103], and such events are seen in the case of white dwarfs[104]. However, for accretion onto a neutron star, we would usually expect to observe a relatively soft outburst (e.g. in the model of[103] the temperature is ~ 10 keV). The spectrum we observe for GRB 230307A consists of evolving synchrotron emission which does not contain a thermal component and is inconsistent with directly observing heating accreting material. Indeed, even for near direct accretion, it is not clear how such a spectrum would be formed in an accreting neutron star scenario. Indeed, in this case, the evolution to very low temperatures on a timescale of ~ 60 days would also not be natural.

Hence, we conclude that no known Galactic systems could explain the observed properties of GRB 230307A.

SI.4.5 GRB 230307A as a white-dwarf – neutron star merger

A final alternative is that GRB 230307A is related to the merger of a white dwarf and a neutron star. Although this is still a "compact object merger", such mergers are very different from those of neutron stars with another neutron star or a black hole. In particular, simulations show that no *r*-process material is produced[105], and so we should not expect the very red emission. Although there are suggestions that GRB 211211A could have been produced by a WD-NS merger[46,106], it is unclear if these could readily explain the detailed spectrophotometric evolution of GRB 230307A.

White dwarf neutron star mergers are appealing, because the long-duration of the gamma-ray emission could suggest a less compact remnant merger event. The wider separation of the binary at the disruption of the white dwarf produces disk accretions times from 100 to 1000s, matching the long duration of these bursts[107]. However, we expect the accretion rate in the mergers to be low, producing less-powerful, and hence, less luminous GRBs. Current WD-NS merger simulations predict a range of light curves that span the emission from GRB 230307A. A Ca feature does exist that is close to the observed line feature, but the models are too blue to explain the shape of the spectra[105]. This is because WD-NS mergers do not produce elements much heavier than the iron peak elements. As we have noticed in matching kilonova models, these elements do not have strong lines beyond $\sim 20{,}000$ Å. The subsequent emission above these wavelengths is very weak in these models. In addition, WD-NS mergers are expected to have fairly week kicks to ensure that the binary remains bound and these mergers are expected to have much lower offsets than neutron star mergers. However, the mass of the best-candidate host galaxy is sufficiently low that the observed offset for this burst can be attained[108].

Therefore to explain the temporal and spectral evolution of the transient in terms of a white dwarf – neutron star merger would require the reddenning at ~ 10 days to be attributed to the early creation of dust, and the later observations with JWST to be caused by a combination of dust (to provide the opacity) and molecular-line emission (to create the observed emission feature at 2.1 microns), see[109]. The details of dust emission are poorly understood, even in well observed supernovae, and depend sensitively on the chemical and kinematic properties of the ejecta and any circumstellar environment, which may be very different in these mergers to normal supernovae. Therefore we do not currently have the necessary models to make strong statements regarding this possibility, although any such model would, by necessity also make an event with strong similarities to AT2017gfo given the comparable observational evolution seen here.

Telescope/filter	Magnitude	Uncertainty
GALEX/FUV	20.87	0.15
GALEX/NUV	20.38	0.13
UVOT/u	20.09	0.30
LS/g	18.28	0.02
LS/r	17.80	0.03
LS/z	17.34	0.05
JWST/$F070W$	17.98	0.01
JWST/$F115W$	17.49	0.01
JWST/$F150W$	17.32	0.01
JWST/$F277W$	17.75	0.01
JWST/$F356W$	17.93	0.01
JWST/$F444W$	18.24	0.01
WISE/$W1$	17.86	0.05
WISE/$W2$	18.23	0.12

Supplementary Table 2. Photometry (in the AB photometric system) of the host galaxy of GRB 230307A. No reddening correction was applied. Uncertainties are given at the 1σ level and are statistical only.

Parameter	Prior bounds	Median & 90% C. I.
$\log(E_K/\mathrm{erg})$	$(49.5, 55)$	$50.7^{+1.0}_{-1.2}$
$\log(n/\mathrm{cm}^{-3})$	(-6,2)	$-0.6^{+2.0}_{-2.0}$
θ_j/rad	(0.01,0.5)	$0.23^{+0.12}_{-0.14}$
$\log(\Gamma_0)$	$(2, 4)$	3 ± 1
$\log(\xi_N)$	$(-2, 0)$	$-1.5^{+0.8}_{-0.5}$
$\log(\varepsilon_e)$	$(-4, -0.5)$	$-1.2^{+0.6}_{-2.2}$
$\log(\varepsilon_B)$	$(-6, -0.5)$	$-2.2^{+1.4}_{-2.0}$
p	$(2.01, 2.99)$	$2.39^{+0.42}_{-0.25}$
$\log(M_{ej}/M_\odot)$	$(-4, 0)$	$-1.23^{+0.57}_{-1.4}$
$\log(v_{ej}/c)$	$(-3, -0.3)$	$-1.0^{+0.51}_{-0.74}$
$\log(\kappa/\mathrm{cm}^2\,\mathrm{g}^{-1})$	$(-1, 2)$	$-0.17^{+1.1}_{-0.73}$
δ	$(1.5, 7)$	$4.4^{+2.4}_{-2.6}$
$\log(f_{sys})$	$(-4, 0)$	$-0.25^{+0.12}_{-0.09}$

Supplementary Table 3. Light curve model parameters, priors and posterior medians and 90% credible intervals.

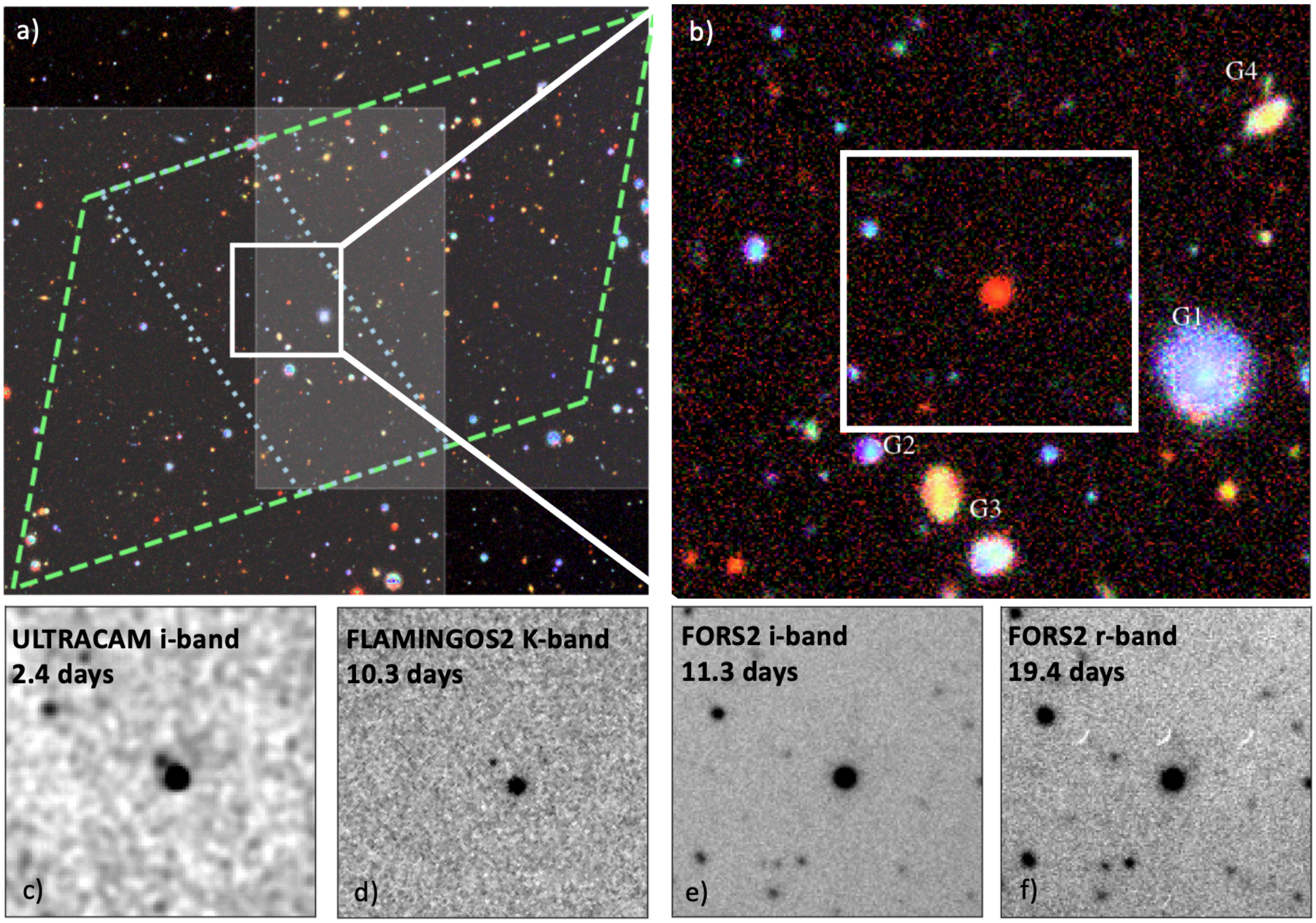

Supplementary Figure 1. Ground based optical and IR imaging of the afterglow of GRB 230307A. Panel (a) shows a legacy survey image of the field, the green trapezoid shows the IPN error box at the time of afterlgow discovery, with the blue dotted lines showing its ultimate refinement. The shaded regions represent observations taken with ULTRACAM 1.4 days post burst. Panel (b) shows the same image, zoomed in to a region around the afterglow. The red source in the centre is an unrelated foreground star (confirmed both by its measured proper motion and spectrum), and several other galaxies can also be seen. Panel (c) shows an ULTRACAM image, where the afterglow can be seen to the north-east of the star. The remaining panels (d,e,f) show the afterglow imaged on $\sim 10-20$ day timescales. At 10 days the source is undetected in deep FORS2 i-band imaging, but well detected in the K-band with Gemini-South. This very red colour was suggestive of an additional component over and above any afterglow, and motivated further follow-up.

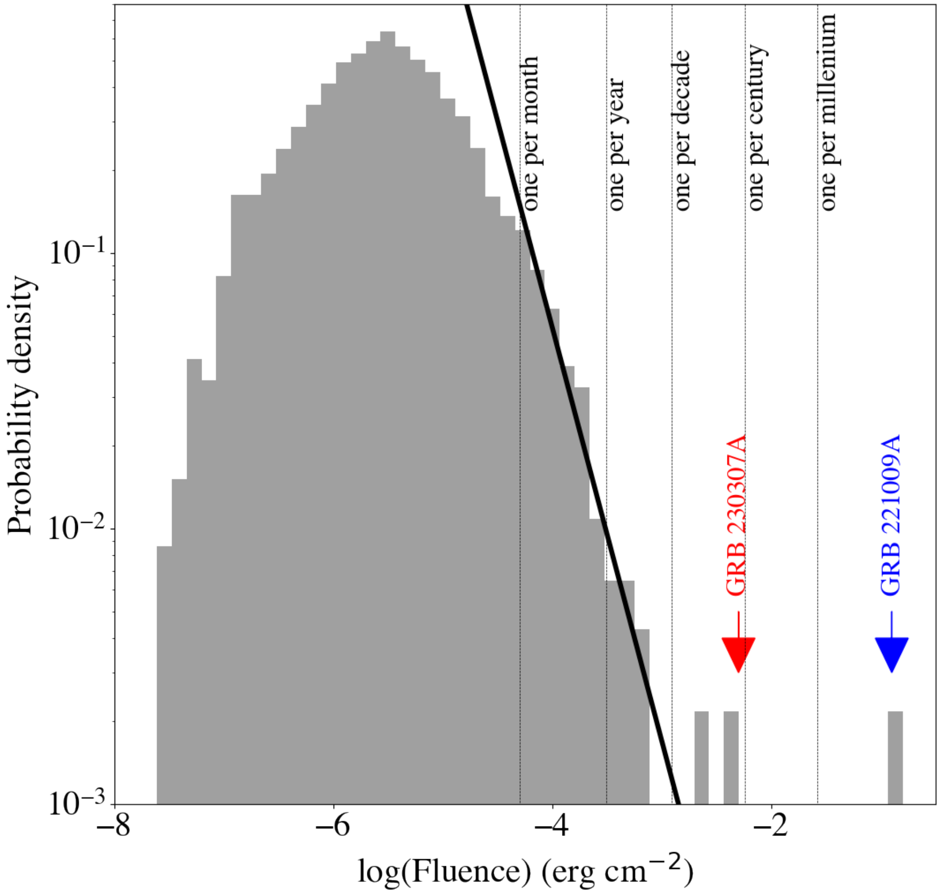

Supplementary Figure 2. The distribution of measure fluence from the *Fermi*/GBM catalogue[7] . The solid line shows an expected slope of $-3/2$ for a uniform distribution. The faint end deviates from this line because of incompleteness. At the brighter end, there are three bursts which appear to be extremely rare, GRB 130427A, GRB 221009A[1] and GRB 230307A. To indicate the apparent rarity we also plot lines representing the expected frequency of events under the assumption of a $-3/2$ slope. We would expect to observe bursts akin to GRB 230307A only once per several decades.

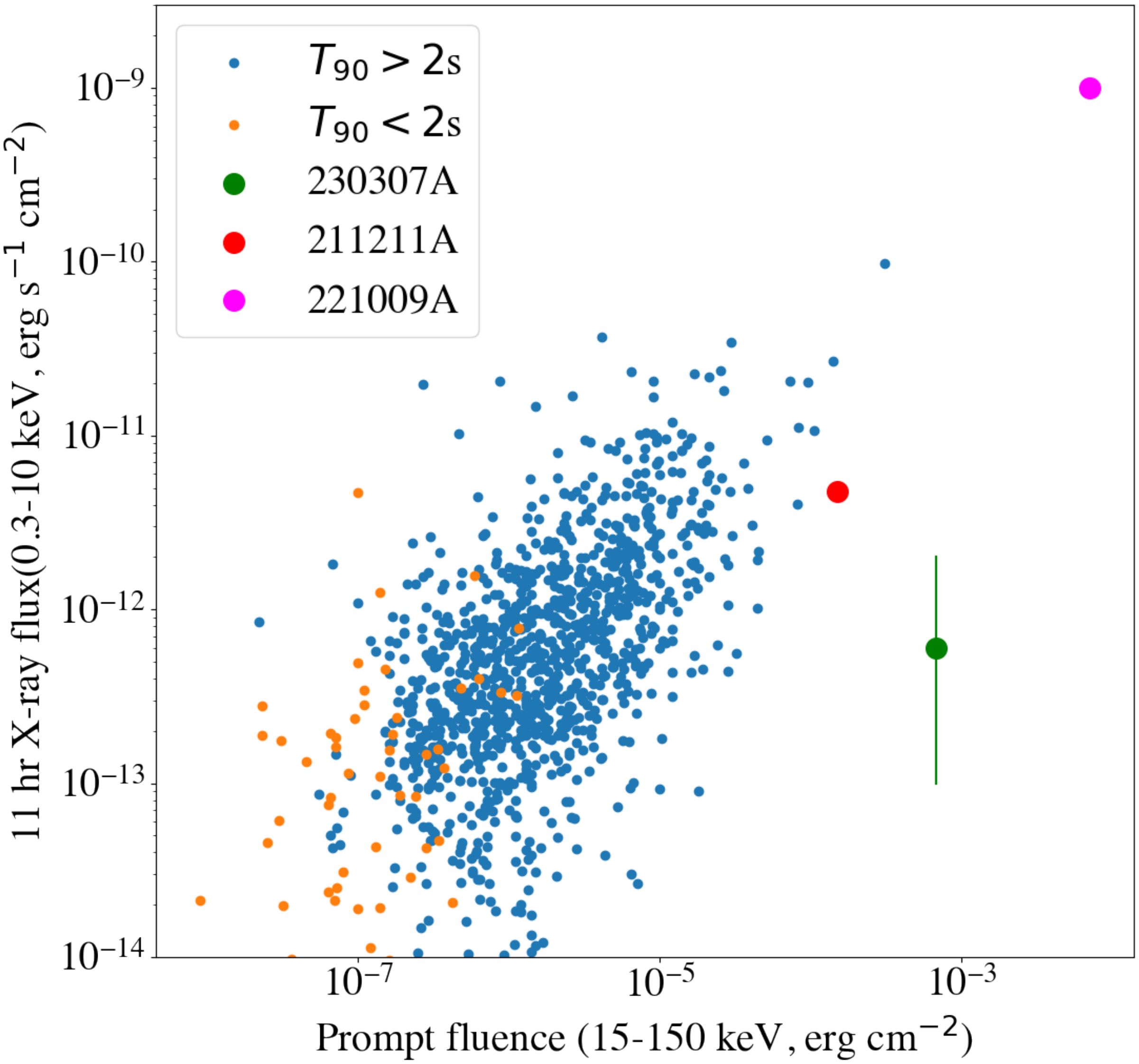

Supplementary Figure 3. A comparison between the prompt fluence and X-ray afterglow flux. The gamma-ray fluence is plotted in the 15-150 keV band, while the X-ray flux is at 11 hours for *Swift* GRBs with GRB 221009A and GRB 230307A added, updated from[9,10]. Error bars (where plotted) are at 1σ. The general 1:1 trend between the prompt fluence and X-ray brightness can clearly be seen, although it has a significant scatter, although a very rare event, GRB 221009A apparently lies on the same relation. However, GRB 211211A and GRB 230307A are clearly outliers to this relation with GRB 230307A occupying a region devoid of other GRBs. This very faint afterglow compared to the prompt emission may be related to a location at large projected offset from its host galaxy in which the density of the ambient medium is very low.

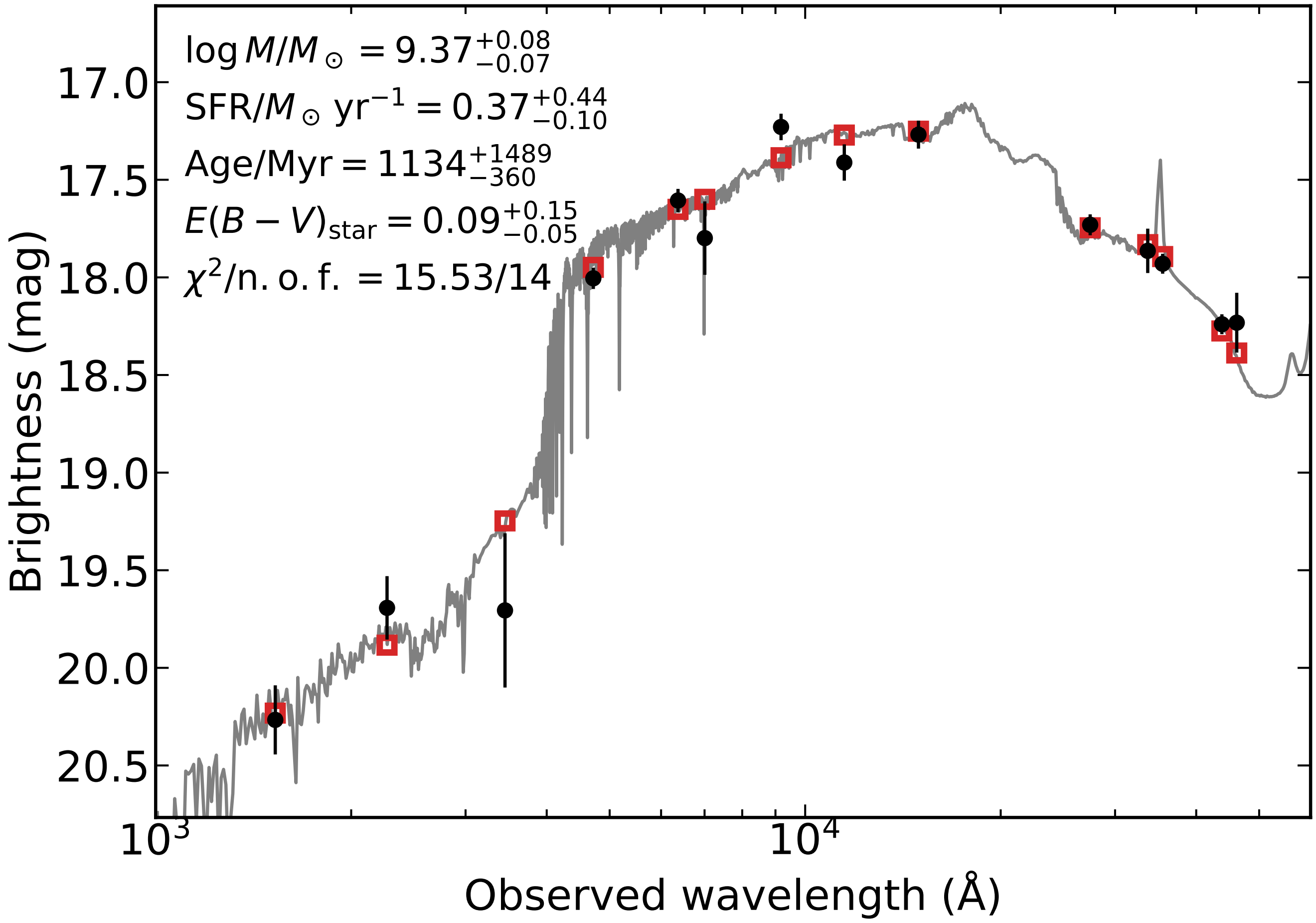

Supplementary Figure 4. Spectral energy distribution (SED) of the host galaxy of GRB 230307A. The SED is plotted from 1000 to 60,000 Å (black data points) and its best fit with the *prospector* SED fitting code (grey shaded curve). In the top right, we also report the values of the model parameters and their 1σ uncertainties. The red squares represent the model-predicted magnitudes. The fitting parameters are shown in the upper-left corner. The abbreviation 'n.o.f.' stands for the number of filters. Error bars are at 1σ.

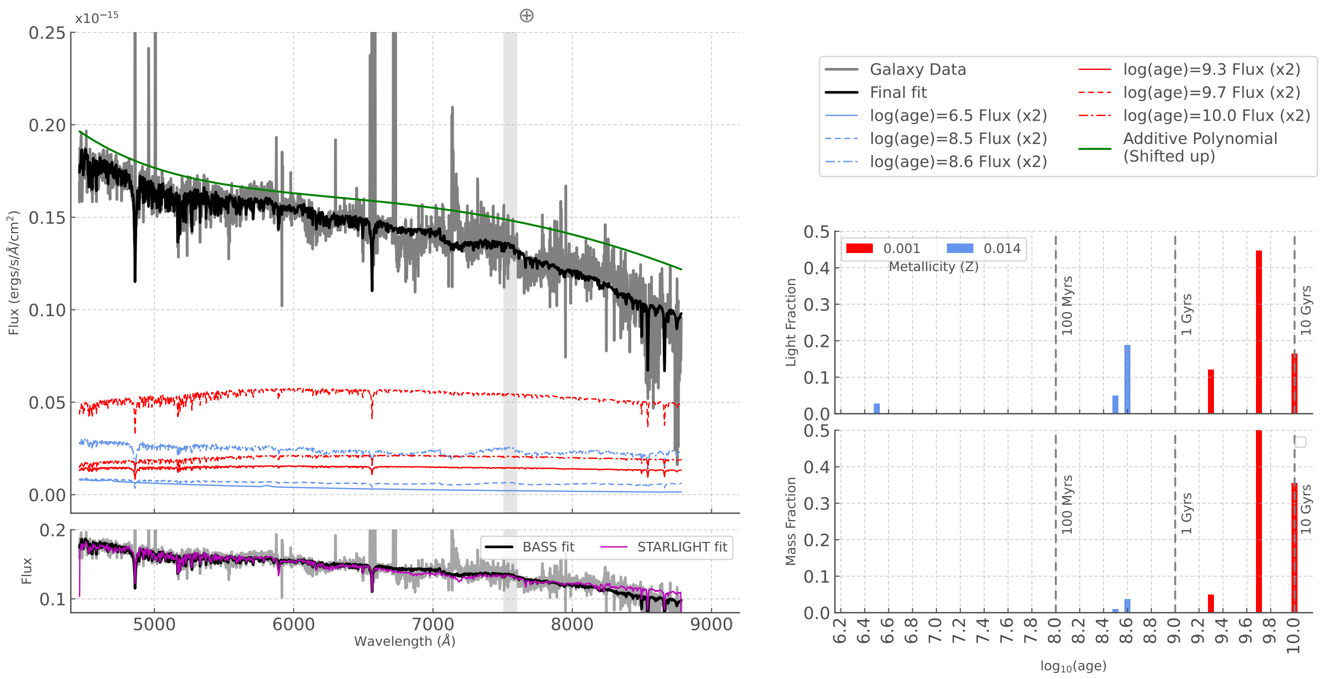

Supplementary Figure 5. Physical properties of the host galaxy of GRB 230307A. SED fit (top left panel) and Star Formation History (right hand panel of the host galaxy obtained with BPASSv.2.2.2–`hoki` templates fit with `ppxf`. We also include a comparison of the final fits obtained with BPASS (2 metallicities) and STALRIGHT (6 metallicites) in the bottom left panel to highlight how similar both fits are.

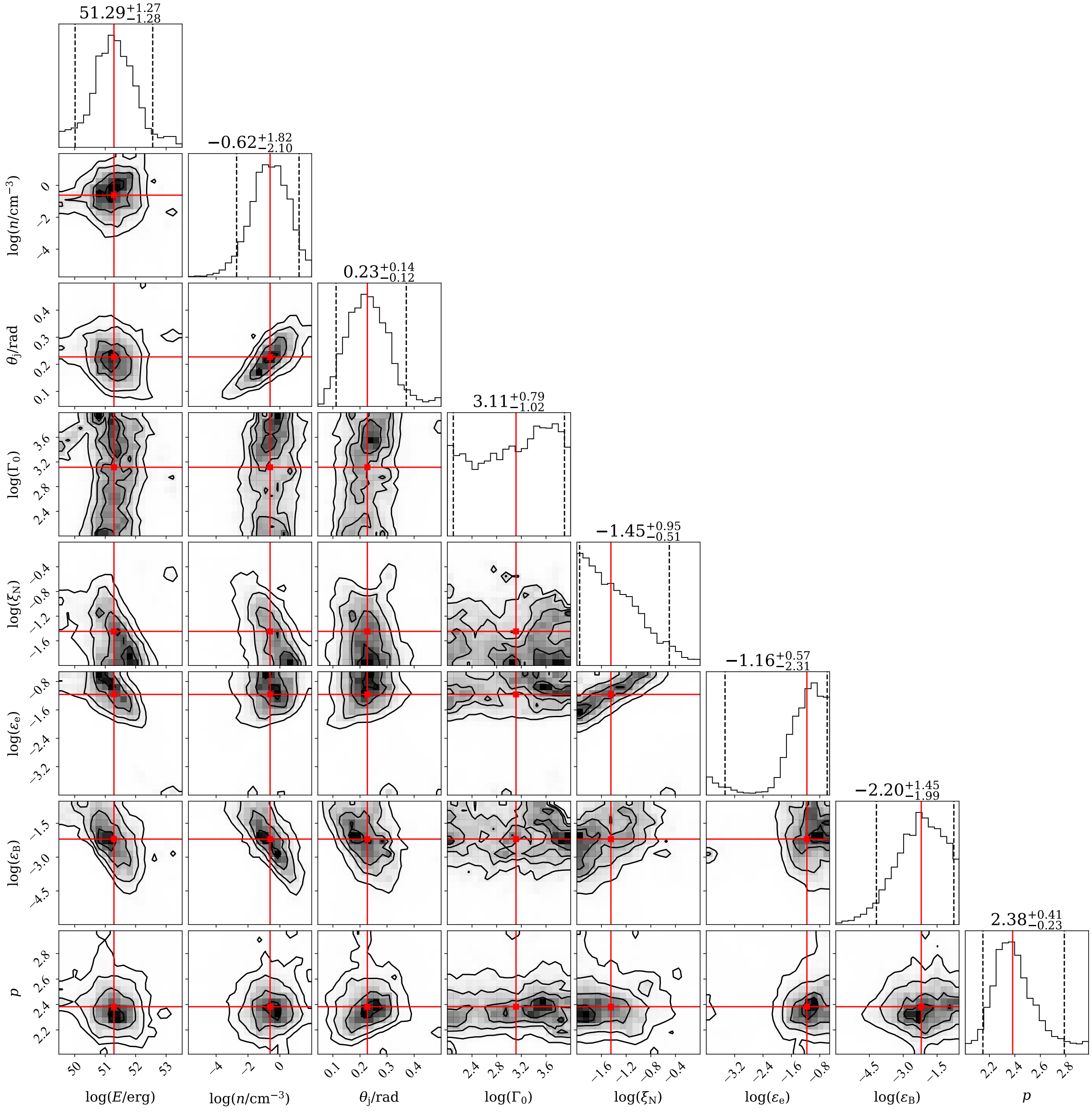

Supplementary Figure 6. **Corner plot of posterior probability density from multi-wavelength light curve fitting**. In this case we have limited the parameters to the relativistic jet afterglow parameter space. Histograms on the diagonal show the marginalized posterior probability densities on the parameters constructed from our MCMC posterior samples. Dashed black lines show the 90% credible interval, while red lines show the medians. The remaining plots show the one, two and three sigma equivalent contours of the joint posterior probability densities of parameter pairs. Red lines and squares mark the medians.

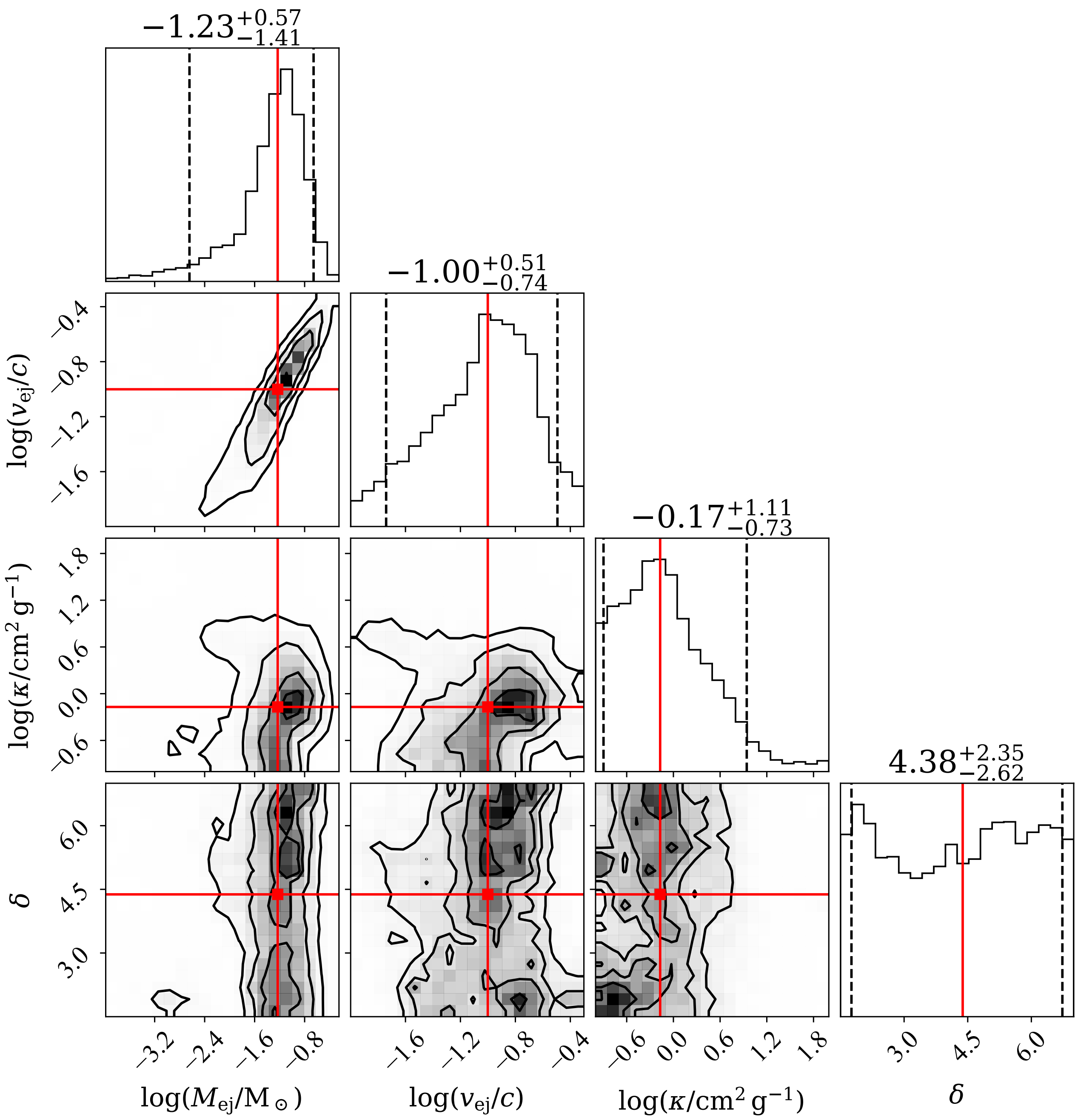

Supplementary Figure 7. Corner plot of posterior probability density from multi-wavelength light curve fitting. In this plot we have limited to the kilonova parameter space such that the figure is similar to figure 6, but for the kilonova model parameters.

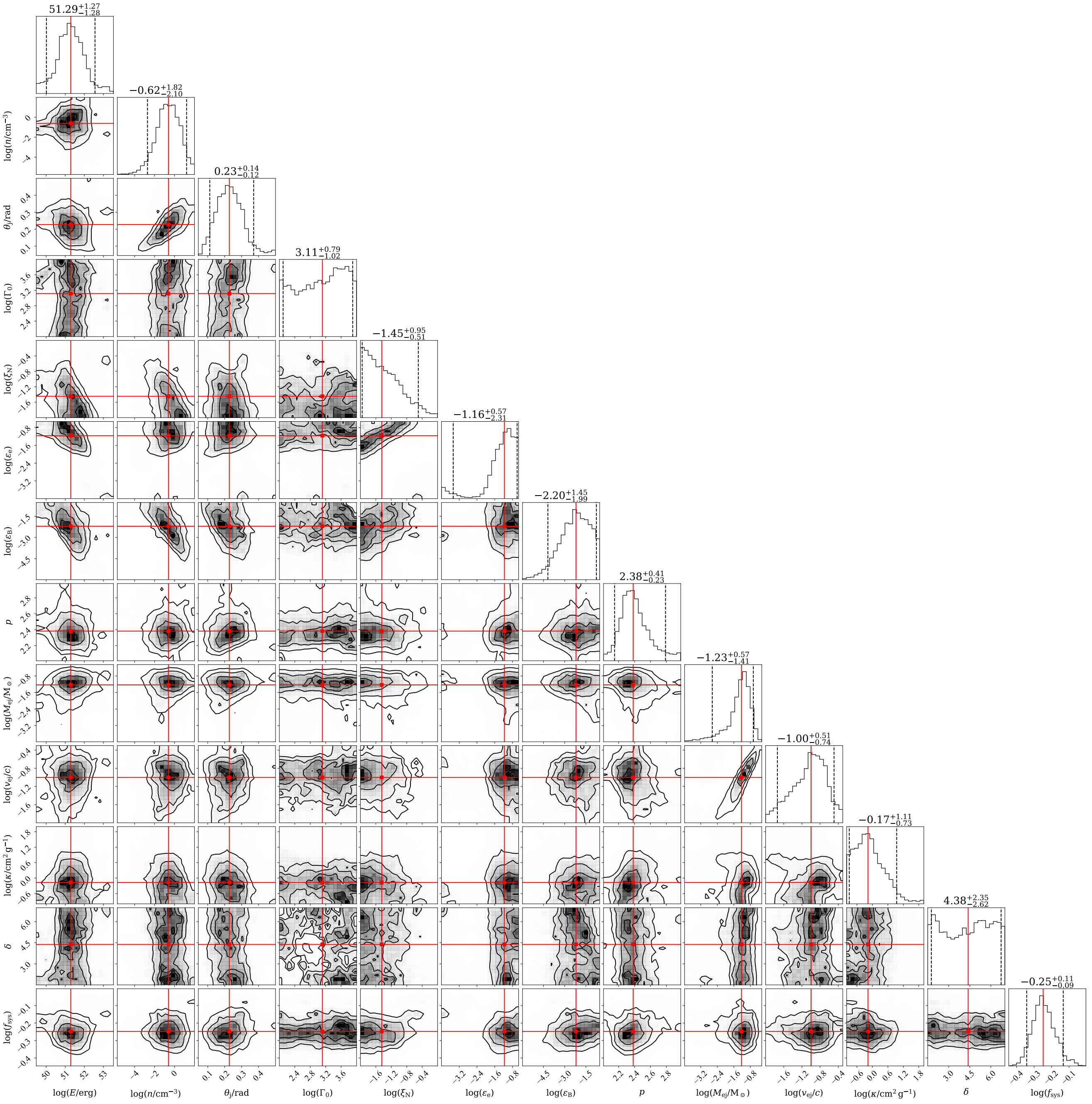

Supplementary Figure 8. Corner plot of posterior probability density from multi-wavelength light curve fitting. Similar to figures 6 and 7, but showing all model parameters, including the nuisance parameter $f_{\rm sys}$.

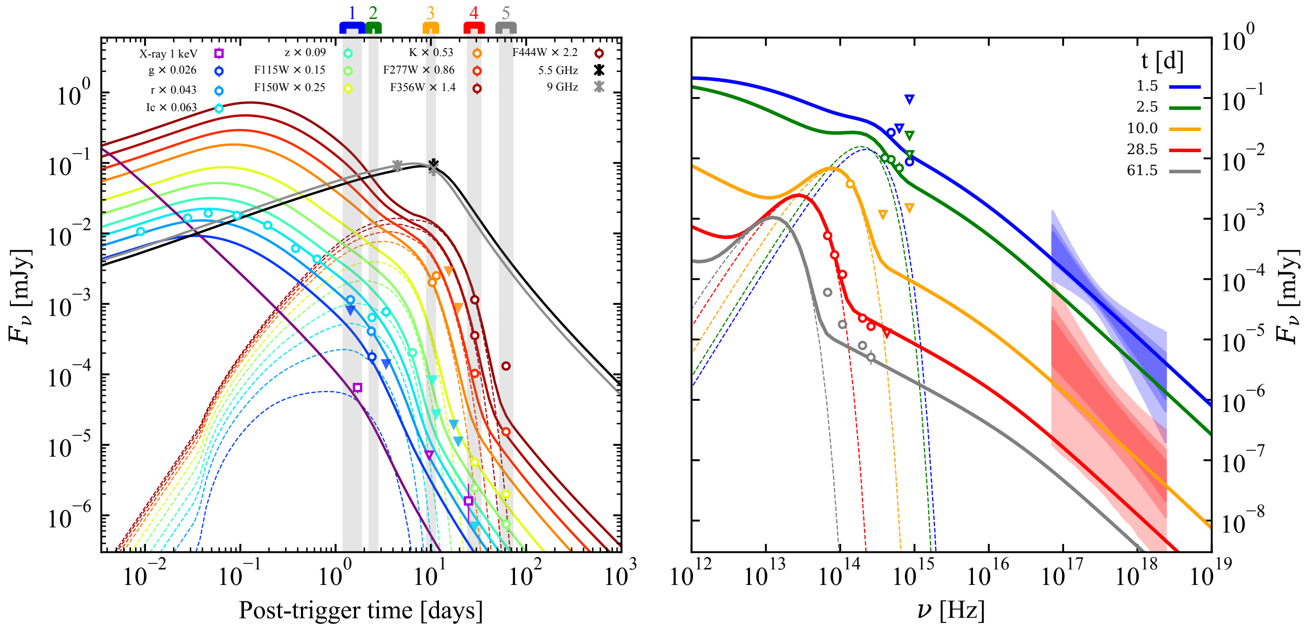

Supplementary Figure 9. Multi-wavelength light curves and model predictions. Markers in the figure show the observed flux density at the position of GRB 230307A in various bands (see legend in the left-hand panel) and at various times. Downward-facing triangles represent upper limits. The optical and near infrared flux densities are multiplied by the numbers reported in the legend for presentation purposes. The butterfly-shaped filled regions in the right-hand panel encompass flux densities consistent at one, two and three sigma (progressively lighter shades) with the *Swift*/XRT and Chandra detections in the 0.3-10 keV band, according to our analysis and adopting a uniform prior on the flux. Solid lines of the corresponding colours show the predicted light curves (left-hand panel) and spectra (right-hand panel) of our afterglow (forward shock only) plus kilonova model at the central frequencies of the bands. Dashed lines single out the contribution of the kilonova.

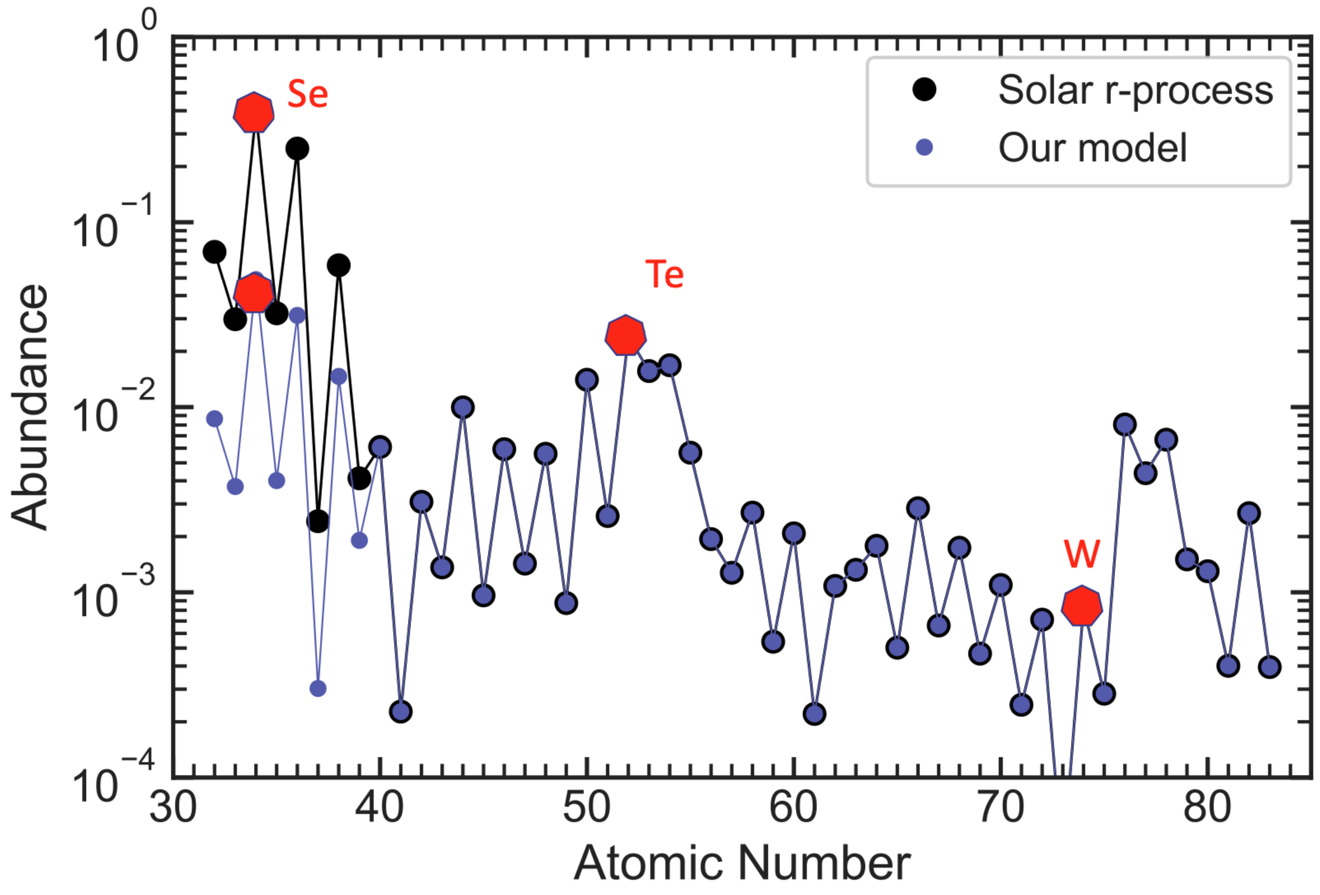

Supplementary Figure 10. Abundances used in the spectral modeling. The abundance is chosen based on the solar r-process residuals. The abundance of the "light" elements ($A < 85$) is reduced relative to the solar pattern. The locations of selenium (Se), tellurium (Te) and tungsten (W) are marked.

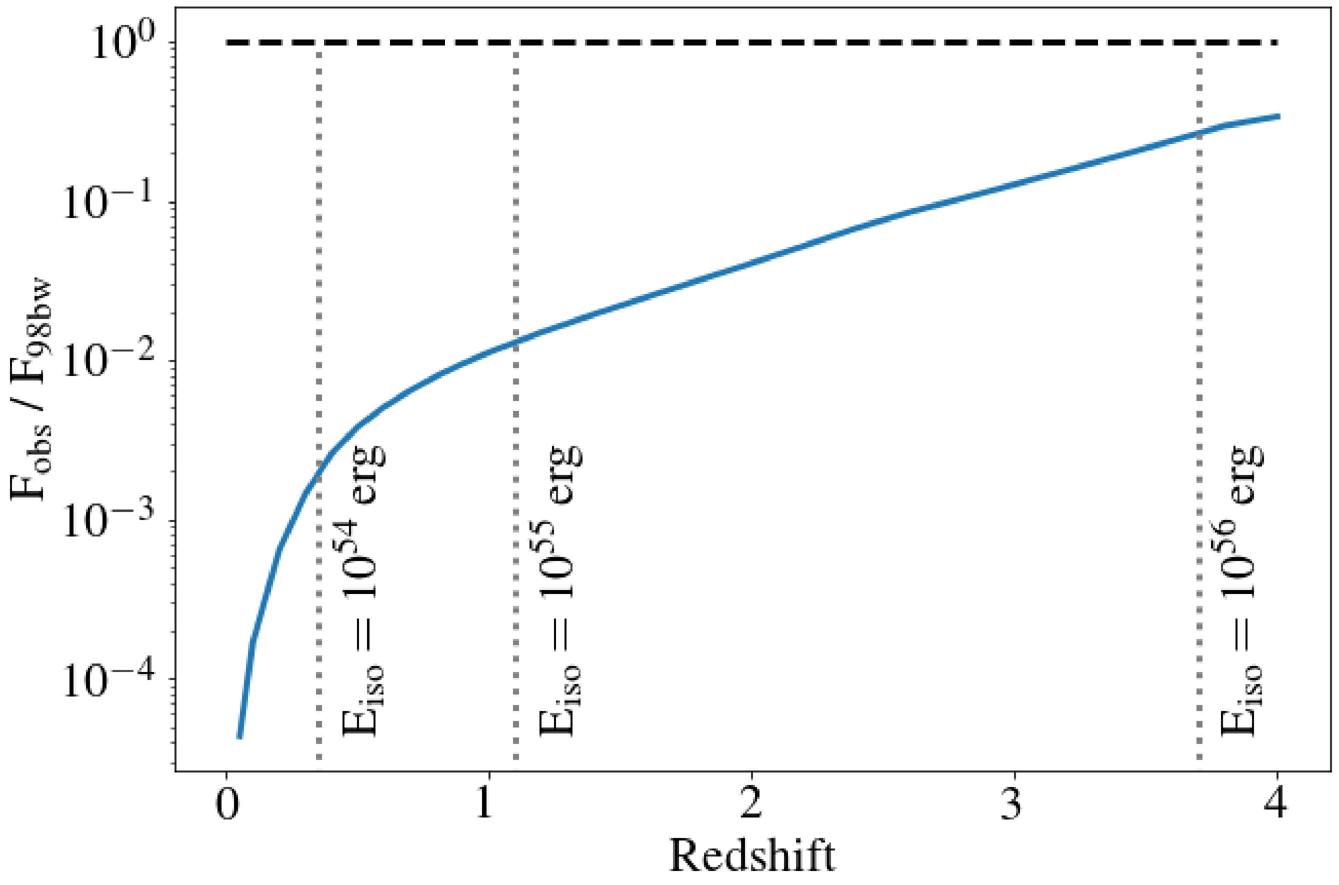

Supplementary Figure 11. Limits on supernova similar to SN 1998bw as a function of redshift. These limits are based on the most constraining detection with *JWST*. At any redshift for which GRB 230307A would not be the most energetic GRB ever observed, any supernova is at least a factor of $\sim$ 100 fainter than SN 1998bw.

References

1. Burns, E. *et al.* GRB 221009A, The BOAT. *arXiv e-prints* arXiv:2302.14037 (2023).
2. Rastinejad, J. C. *et al.* A kilonova following a long-duration gamma-ray burst at 350 Mpc. *Nature* **612**, 223–227 (2022).
3. Dalessi, S., Roberts, O. J., Meegan, C. & Fermi GBM Team. GRB 230307A: Fermi GBM Observation of a very bright burst. *GRB Coordinates Network* **33411**, 1 (2023).
4. Mangan, J., Dunwoody, R., Meegan, C. & Fermi GBM Team. GRB 211211A: Fermi GBM observation. *GRB Coordinates Network* **31210**, 1 (2021).
5. Gompertz, B. P. *et al.* The case for a minute-long merger-driven gamma-ray burst from fast-cooling synchrotron emission. *Nature Astronomy* **7**, 67–79 (2023).
6. Wang, Y., Xia, Z.-Q., Zheng, T.-C., Ren, J. & Fan, Y.-Z. A Broken "α-intensity" Relation Caused by the Evolving Photosphere Emission and the Nature of the Extraordinarily Bright GRB 230307A. *Astrophys. J. Lett.* **953**, L8 (2023).
7. von Kienlin, A. *et al.* The Fourth Fermi-GBM Gamma-Ray Burst Catalog: A Decade of Data. *Astrophys. J.* **893**, 46 (2020).
8. Troja, E. *et al.* A nearby long gamma-ray burst from a merger of compact objects. *Nature* **612**, 228–231 (2022).
9. Gehrels, N., Ramirez-Ruiz, E. & Fox, D. B. Gamma-Ray Bursts in the Swift Era. *Annu. Rev. Astron. Astrophys.* **47**, 567–617 (2009).
10. Nysewander, M., Fruchter, A. S. & Pe'er, A. A Comparison of the Afterglows of Short- and Long-duration Gamma-ray Bursts. *Astrophys. J.* **701**, 824–836 (2009).
11. Evans, P. A. *et al.* The Swift Burst Analyser. I. BAT and XRT spectral and flux evolution of gamma ray bursts. *Astron. Astrophys.* **519**, A102 (2010).
12. Lien, A. *et al.* The Third Swift Burst Alert Telescope Gamma-Ray Burst Catalog. *Astrophys. J.* **829**, 7 (2016).
13. Goldstein, A. *et al.* An Ordinary Short Gamma-Ray Burst with Extraordinary Implications: Fermi-GBM Detection of GRB 170817A. *Astrophys. J. Lett.* **848**, L14 (2017).
14. Hajela, A. *et al.* Evidence for X-Ray Emission in Excess to the Jet-afterglow Decay 3.5 yr after the Binary Neutron Star Merger GW 170817: A New Emission Component. *Astrophys. J. Lett.* **927**, L17 (2022).
15. Schulze, S. *et al.* GRB 120422A/SN 2012bz: Bridging the gap between low- and high-luminosity gamma-ray bursts. *Astron. Astrophys.* **566**, A102 (2014).
16. Fausnaugh, M. M. *et al.* Observations of GRB 230307A by TESS. *arXiv e-prints* arXiv:2303.07319 (2023).
17. Gillanders, J., O'Connor, B., Dichiara, S. & Troja, E. GRB 230307A: Continued Gemini-South observations confirm rapid optical fading. *GRB Coordinates Network* **33485**, 1 (2023).
18. Bloom, J. S., Kulkarni, S. R. & Djorgovski, S. G. The Observed Offset Distribution of Gamma-Ray Bursts from Their Host Galaxies: A Robust Clue to the Nature of the Progenitors. *Astron. J.* **123**, 1111–1148 (2002).
19. Straatman, C. M. S. *et al.* The Sizes of Massive Quiescent and Star-forming Galaxies at $z \sim 4$ with ZFOURGE and CANDELS. *Astrophys. J. Lett.* **808**, L29 (2015).
20. Windhorst, R. A. *et al.* JWST PEARLS. Prime Extragalactic Areas for Reionization and Lensing Science: Project Overview and First Results. *Astron. J.* **165**, 13 (2023).
21. Blanchard, P. K., Berger, E. & Fong, W.-f. The Offset and Host Light Distributions of Long Gamma-Ray Bursts: A New View From HST Observations of Swift Bursts. *Astrophys. J.* **817**, 144 (2016).
22. Krühler, T. *et al.* GRB hosts through cosmic time. VLT/X-Shooter emission-line spectroscopy of 96 γ-ray-burst-selected galaxies at $0.1 < z < 3.6$. *Astron. Astrophys.* **581**, A125 (2015).
23. Martin, D. C. *et al.* The Galaxy Evolution Explorer: A Space Ultraviolet Survey Mission. *Astrophys. J.* **619**, L1 (2005).
24. Dey, A. *et al.* Overview of the DESI Legacy Imaging Surveys. *Astron. J.* **157**, 168 (2019).
25. Wright, E. L. *et al.* The Wide-field Infrared Survey Explorer (WISE): Mission Description and Initial On-orbit Performance. *Astron. J.* **140**, 1868–1881 (2010).
26. Lang, D. unWISE: Unblurred Coadds of the WISE Imaging. *Astron. J.* **147**, 108 (2014).
27. Mainzer, A. *et al.* Initial Performance of the NEOWISE Reactivation Mission. *Astrophys. J.* **792**, 30 (2014).

28. Meisner, A. M., Lang, D. & Schlegel, D. J. Full-depth Coadds of the WISE and First-year NEOWISE-reactivation Images. *Astron. J.* **153**, 38 (2017).

29. Wright, A. H. *et al.* Galaxy And Mass Assembly: accurate panchromatic photometry from optical priors using LAMBDAR. *Mon. Not. R. Astron. Soc.* **460**, 765 (2016).

30. Schulze, S. *et al.* The Palomar Transient Factory Core-collapse Supernova Host-galaxy Sample. I. Host-galaxy Distribution Functions and Environment Dependence of Core-collapse Supernovae. *Astrophys. J. Suppl. Ser.* **255**, 29 (2021).

31. Johnson, B. D., Leja, J., Conroy, C. & Speagle, J. S. Stellar Population Inference with Prospector. *Astrophys. J. Suppl. Ser.* **254**, 22 (2021).

32. Conroy, C. & Gunn, J. E. FSPS: Flexible Stellar Population Synthesis. Astrophysics Source Code Library, record ascl:1010.043 (2010). 1010.043.

33. Calzetti, D. *et al.* The Dust Content and Opacity of Actively Star-forming Galaxies. *Astrophys. J.* **533**, 682–695 (2000).

34. Ferland, G. J. *et al.* The 2017 Release Cloudy. *Rev. Mexicana Astron. Astrofis.* **53**, 385–438 (2017).

35. Speagle, J. S. DYNESTY: a dynamic nested sampling package for estimating Bayesian posteriors and evidences. *Mon. Not. R. Astron. Soc.* **493**, 3132–3158 (2020).

36. Eldridge, J. J. *et al.* Binary Population and Spectral Synthesis Version 2.1: Construction, Observational Verification, and New Results. *Publ. Astron. Soc. Aust.* **34**, e058 (2017).

37. Stanway, E. R. & Eldridge, J. J. Re-evaluating old stellar populations. *Mon. Not. R. Astron. Soc.* **479**, 75–93 (2018).

38. Stevance, H., Eldridge, J. & Stanway, E. Hoki: Making BPASS accessible through Python. *The Journal of Open Source Software* **5**, 1987 (2020).

39. Cappellari, M. Improving the full spectrum fitting method: accurate convolution with Gauss-Hermite functions. *Mon. Not. R. Astron. Soc.* **466**, 798–811 (2017).

40. Stevance, H. F. *et al.* End-to-end study of the host galaxy and genealogy of the first binary neutron star merger. *Nature Astronomy* **7**, 444–450 (2023).

41. Cid Fernandes, R., Mateus, A., Sodré, L., Stasińska, G. & Gomes, J. M. Semi-empirical analysis of Sloan Digital Sky Survey galaxies - I. Spectral synthesis method. *Mon. Not. R. Astron. Soc.* **358**, 363–378 (2005).

42. Bruzual, G. & Charlot, S. Stellar population synthesis at the resolution of 2003. *Mon. Not. R. Astron. Soc.* **344**, 1000–1028 (2003).

43. Chabrier, G. Galactic Stellar and Substellar Initial Mass Function. *Publ. Astron. Soc. Pac.* **115**, 763–795 (2003).

44. Kennicutt, J., Robert C. The Global Schmidt Law in Star-forming Galaxies. *Astrophys. J.* **498**, 541–552 (1998).

45. Marino, R. A. *et al.* The O3N2 and N2 abundance indicators revisited: improved calibrations based on CALIFA and T_e-based literature data. *Astron. Astrophys.* **559**, A114 (2013).

46. Yang, J. *et al.* A long-duration gamma-ray burst with a peculiar origin. *Nature* **612**, 232–235 (2022).

47. Fulton, M. D. *et al.* The Optical Light Curve of GRB 221009A: The Afterglow and the Emerging Supernova. *Astrophys. J. Lett.* **946**, L22 (2023).

48. Shrestha, M. *et al.* Limit on Supernova Emission in the Brightest Gamma-Ray Burst, GRB 221009A. *Astrophys. J. Lett.* **946**, L25 (2023).

49. Levan, A. J. *et al.* The First JWST Spectrum of a GRB Afterglow: No Bright Supernova in Observations of the Brightest GRB of all Time, GRB 221009A. *Astrophys. J. Lett.* **946**, L28 (2023).

50. Srinivasaragavan, G. P. *et al.* A Sensitive Search for Supernova Emission Associated with the Extremely Energetic and Nearby GRB 221009A. *Astrophys. J. Lett.* **949**, L39 (2023).

51. Blanchard, P. K. *et al.* JWST Observations of the Extraordinary GRB 221009A Reveal an Ordinary Supernova Without Signs of *r*-Process Enrichment in a Low-Metallicity Galaxy. *arXiv e-prints* arXiv:2308.14197 (2023).

52. Yin, Y.-H. I. *et al.* GRB 211211A-like Events and How Gravitational Waves May Tell Their Origins. *Astrophys. J. Lett.* **954**, L17 (2023).

53. Yang, B. *et al.* A possible macronova in the late afterglow of the long-short burst GRB 060614. *Nature Communications* **6**, 7323 (2015).

54. Jin, Z.-P. *et al.* The Light Curve of the Macronova Associated with the Long-Short Burst GRB 060614. *Astrophys. J. Lett.* **811**, L22 (2015).

55. Liang, E., Zhang, B., Virgili, F. & Dai, Z. G. Low-Luminosity Gamma-Ray Bursts as a Unique Population: Luminosity Function, Local Rate, and Beaming Factor. *Astrophys. J.* **662**, 1111–1118 (2007).

56. Salafia, O. S. *et al.* Multiwavelength View of the Close-by GRB 190829A Sheds Light on Gamma-Ray Burst Physics. *Astrophys. J. Lett.* **931**, L19 (2022).

57. Mei, A. *et al.* Gigaelectronvolt emission from a compact binary merger. *Nature* **612**, 236–239 (2022).

58. Hotokezaka, K. & Nakar, E. Radioactive Heating Rate of r-process Elements and Macronova Light Curve. *Astrophys. J.* **891**, 152 (2020).

59. Panaitescu, A. & Kumar, P. Analytic Light Curves of Gamma-Ray Burst Afterglows: Homogeneous versus Wind External Media. *Astrophys. J.* **543**, 66–76 (2000).

60. Foreman-Mackey, D., Hogg, D. W., Lang, D. & Goodman, J. emcee: The mcmc hammer. *PASP* **125**, 306–312 (2013).

61. Goodman, J. & Weare, J. Ensemble samplers with affine invariance. *Communications in Applied Mathematics and Computational Science* **5**, 65–80 (2010).

62. Perley, D. A. *et al.* GRB 080503: Implications of a Naked Short Gamma-Ray Burst Dominated by Extended Emission. *Astrophys. J.* **696**, 1871–1885 (2009).

63. Fong, W. *et al.* Short GRB 130603B: Discovery of a Jet Break in the Optical and Radio Afterglows, and a Mysterious Late-time X-Ray Excess. *Astrophys. J.* **780**, 118 (2014).

64. Gillanders, J. H., Sim, S. A., Smartt, S. J., Goriely, S. & Bauswein, A. Modelling the spectra of the kilonova AT2017gfo – II: Beyond the photospheric epochs. *arXiv e-prints* arXiv:2306.15055 (2023).

65. Kasen, D., Badnell, N. R. & Barnes, J. Opacities and Spectra of the r-process Ejecta from Neutron Star Mergers. *Astrophys. J.* **774**, 25 (2013).

66. Tanaka, M. & Hotokezaka, K. Radiative Transfer Simulations of Neutron Star Merger Ejecta. *Astrophys. J.* **775**, 113 (2013).

67. Fontes, C. J., Fryer, C. L., Hungerford, A. L., Wollaeger, R. T. & Korobkin, O. A line-binned treatment of opacities for the spectra and light curves from neutron star mergers. *Mon. Not. R. Astron. Soc.* **493**, 4143–4171 (2020).

68. Takami, H., Nozawa, T. & Ioka, K. Dust Formation in Macronovae. *Astrophys. J. Lett.* **789**, L6 (2014).

69. Hotokezaka, K., Tanaka, M., Kato, D. & Gaigalas, G. Tungsten versus Selenium as a potential source of kilonova nebular emission observed by Spitzer. *Mon. Not. R. Astron. Soc.* **515**, L89–L93 (2022).

70. Kramida, A., Yu. Ralchenko, Reader, J. & and NIST ASD Team. NIST Atomic Spectra Database (ver. 5.9), [Online]. Available: `https://physics.nist.gov/asd` [2017, April 9]. National Institute of Standards and Technology, Gaithersburg, MD. (2021).

71. Madonna, S. *et al.* Neutron-capture Elements in Planetary Nebulae: First Detections of Near-infrared [Te III] and [Br V] Emission Lines. *Astrophys. J. Lett.* **861**, L8 (2018).

72. Bar-Shalom, A., Klapisch, M. & Oreg, J. HULLAC, an integrated computer package for atomic processes in plasmas. *J. Quant. Spectrosc. Radiat. Transf.* **71**, 169–188 (2001).

73. Pognan, Q., Jerkstrand, A. & Grumer, J. On the validity of steady-state for nebular phase kilonovae. *Mon. Not. R. Astron. Soc.* **510**, 3806–3837 (2022).

74. Hotokezaka, K., Tanaka, M., Kato, D. & Gaigalas, G. Nebular emission from lanthanide-rich ejecta of neutron star merger. *Mon. Not. R. Astron. Soc.* **506**, 5863–5877 (2021).

75. Hotokezaka, K., Tanaka, M., Kato, D. & Gaigalas, G. Tellurium emission line in kilonova AT 2017gfo. *arXiv e-prints* arXiv:2307.00988 (2023).

76. Barnes, J., Kasen, D., Wu, M.-R. & Martínez-Pinedo, G. Radioactivity and Thermalization in the Ejecta of Compact Object Mergers and Their Impact on Kilonova Light Curves. *Astrophys. J.* **829**, 110 (2016).

77. Waxman, E., Ofek, E. O. & Kushnir, D. Late-time Kilonova Light Curves and Implications to GW170817. *Astrophys. J.* **878**, 93 (2019).

78. Yu, N., Nagourney, W. & Dehmelt, H. Radiative Lifetime Measurement of the Ba^+ Metastable $D_{3/2}$ State. *Phys. Rev. Lett.* **78**, 4898–4901 (1997).

79. Domoto, N. *et al.* Lanthanide Features in Near-infrared Spectra of Kilonovae. *Astrophys. J.* **939**, 8 (2022).

80. Fruchter, A. S. *et al.* Long γ-ray bursts and core-collapse supernovae have different environments. *Nature* **441**, 463–468 (2006).

81. Lyman, J. D. *et al.* The host galaxies and explosion sites of long-duration gamma ray bursts: Hubble Space Telescope near-infrared imaging. *Mon. Not. R. Astron. Soc.* **467**, 1795–1817 (2017).

82. Atteia, J. L. *et al.* The Maximum Isotropic Energy of Gamma-ray Bursts. *Astrophys. J.* **837**, 119 (2017).

83. de Souza, R. S., Yoshida, N. & Ioka, K. Populations III.1 and III.2 gamma-ray bursts: constraints on the event rate for future radio and X-ray surveys. *Astron. Astrophys.* **533**, A32 (2011).

84. Toma, K., Sakamoto, T. & Mészáros, P. Population III Gamma-ray Burst Afterglows: Constraints on Stellar Masses and External Medium Densities. *Astrophys. J.* **731**, 127 (2011).

85. Toma, K., Yoon, S.-C. & Bromm, V. Gamma-Ray Bursts and Population III Stars. *Space Sci. Rev.* **202**, 159–180 (2016).

86. Tolstov, A. *et al.* Multicolor Light Curve Simulations of Population III Core-Collapse Supernovae: From Shock Breakout to ^{56}Co Decay. *Astrophys. J.* **821**, 124 (2016).

87. Ohkubo, T., Nomoto, K., Umeda, H., Yoshida, N. & Tsuruta, S. Evolution of Very Massive Population III Stars with Mass Accretion from Pre-main Sequence to Collapse. *Astrophys. J.* **706**, 1184–1193 (2009).

88. Perley, D. A. *et al.* The fast, luminous ultraviolet transient AT2018cow: extreme supernova, or disruption of a star by an intermediate-mass black hole? *Mon. Not. R. Astron. Soc.* **484**, 1031–1049 (2019).

89. Ho, A. Y. Q. *et al.* A Search for Extragalactic Fast Blue Optical Transients in ZTF and the Rate of AT2018cow-like Transients. *Astrophys. J.* **949**, 120 (2023).

90. Andreoni, I. *et al.* A very luminous jet from the disruption of a star by a massive black hole. *Nature* **612**, 430–434 (2022).

91. Pasham, D. R. *et al.* The Birth of a Relativistic Jet Following the Disruption of a Star by a Cosmological Black Hole. *Nature Astronomy* **7**, 88–104 (2023).

92. Waxman, E., Ofek, E. O. & Kushnir, D. Strong NIR emission following the long duration GRB 211211A: Dust heating as an alternative to a kilonova. *arXiv e-prints* arXiv:2206.10710 (2022).

93. Thomas, R. *et al.* The intergalactic medium transmission towards z $\gtrsim$ 4 galaxies with VANDELS and the impact of dust attenuation. *Astron. Astrophys.* **634**, A110 (2020).

94. Nicholl, M., Guillochon, J. & Berger, E. The Magnetar Model for Type I Superluminous Supernovae. I. Bayesian Analysis of the Full Multicolor Light-curve Sample with MOSFiT. *Astrophys. J.* **850**, 55 (2017).

95. Gillanders, J., O'Connor, B., Dichiara, S. & Troja, E. GRB 230307A: Continued Gemini-South observations confirm rapid optical fading. *GRB Coordinates Network* **33485**, 1 (2023).

96. Zapartas, E. *et al.* Delay-time distribution of core-collapse supernovae with late events resulting from binary interaction. *Astron. Astrophys.* **601**, A29 (2017).

97. Perets, H. B. *et al.* A faint type of supernova from a white dwarf with a helium-rich companion. *Nature* **465**, 322–325 (2010).

98. Agudo, I. *et al.* Panning for gold, but finding helium: Discovery of the ultra-stripped supernova SN 2019wxt from gravitational-wave follow-up observations. *Astron. Astrophys.* **675**, A201 (2023).

99. Fryer, C. L., Young, P. A. & Hungerford, A. L. Explosive Nucleosynthesis from Gamma-Ray Burst and Hypernova Progenitors: Direct Collapse versus Fallback. *Astrophys. J.* **650**, 1028–1047 (2006).

100. Fryer, C. L., Hungerford, A. L. & Young, P. A. Light-Curve Calculations of Supernovae from Fallback Gamma-Ray Bursts. *Astrophys. J. Lett.* **662**, L55–L58 (2007).

101. Palmer, D. M. *et al.* A giant γ-ray flare from the magnetar SGR 1806 - 20. *Nature* **434**, 1107–1109 (2005).

102. Bibby, J. L., Crowther, P. A., Furness, J. P. & Clark, J. S. A downward revision to the distance of the 1806-20 cluster and associated magnetar from Gemini Near-Infrared Spectroscopy. *Mon. Not. R. Astron. Soc.* **386**, L23–L27 (2008).

103. Campana, S. *et al.* The unusual gamma-ray burst GRB 101225A explained as a minor body falling onto a neutron star. *Nature* **480**, 69–71 (2011).

104. Gänsicke, B. T. *et al.* Accretion of a giant planet onto a white dwarf star. *Nature* **576**, 61–64 (2019).

105. Kaltenborn, M. A. *et al.* Abundances and Transients from Neutron Star-White Dwarf Mergers. *arXiv e-prints* arXiv:2209.13061 (2022).

106. Zhong, S.-Q., Li, L. & Dai, Z.-G. GRB 211211A: A Neutron Star-White Dwarf Merger? *Astrophys. J. Lett.* **947**, L21 (2023).

107. Fryer, C. L., Woosley, S. E., Herant, M. & Davies, M. B. Merging White Dwarf/Black Hole Binaries and Gamma-Ray Bursts. *Astrophys. J.* **520**, 650–660 (1999).

108. Toonen, S., Perets, H. B., Igoshev, A. P., Michaely, E. & Zenati, Y. The demographics of neutron star - white dwarf mergers. Rates, delay-time distributions, and progenitors. *Astron. Astrophys.* **619**, A53 (2018).

109. Gillanders, J. H. *et al.* Heavy element nucleosynthesis associated with a gamma-ray burst. *arXiv e-prints* arXiv:2308.00633 (2023).

110. Levan, A. J. *et al.* The First JWST Spectrum of a GRB Afterglow: No Bright Supernova in Observations of the Brightest GRB of all Time, GRB 221009A. *Astrophys. J. Lett.* **946**, L28 (2023).

111. Levan, A. J. *et al.* Hubble Space Telescope Observations of the Afterglow, Supernova, and Host Galaxy Associated with the Extremely Bright GRB 130427A. *Astrophys. J.* **792**, 115 (2014).

112. Melandri, A. *et al.* GRB 171010A/SN 2017htp: a GRB-SN at z = 0.33. *Mon. Not. R. Astron. Soc.* **490**, 5366–5374 (2019).

113. Melandri, A. *et al.* The supernova of the MAGIC gamma-ray burst GRB 190114C. *Astron. Astrophys.* **659**, A39 (2022).

114. Jakobsson, P. *et al.* A mean redshift of 2.8 for Swift gamma-ray bursts. *Astron. Astrophys.* **447**, 897–903 (2006).
